# Delineating phenotypic heterogeneity in human regulatory T cells across developmental stages and therapeutic sources

**DOI:** 10.3389/fimmu.2026.1697723

**Published:** 2026-01-22

**Authors:** Samikshya Santosh Nirmala, Yueyuan Hu, Friederike Dorothea Floegel, Hugo Cruz, Johanna Morgenstern, Alexander Platz, Marcel Vollroth, Anke Fuchs

**Affiliations:** 1 Center for Regenerative Therapies Dresden (CRTD), Center for Molecular and Cellular Bioengineering (CMCB), TUD Dresden University of Technology, Dresden, Germany; 2 DKMS Stem Cell Bank, Dresden, Germany; 3 Institute of Transfusion Medicine and Transplant Engineering, Hannover Medical School, Hannover, Germany; 4 Faculty of Engineering, University of Porto, Porto, Portugal; 5 Pediatric Cardiac Surgery, Heart Center Leipzig, University Hospital Leipzig, Leipzig, Germany

**Keywords:** adult peripheral blood, effector T cells, immunophenotyping, pediatric thymus, regulatory T cells, Treg development, Treg markers, umbilical cord blood

## Abstract

**Background:**

FOXP3^+^ regulatory T cells (Tregs) play a pivotal role in maintaining immune homeostasis and self-tolerance. Despite advances in Treg-based immunosuppressive therapies, precise identification of human Tregs facilitating their isolation with high purity remains challenging because canonical markers such as FOXP3 and CD25 are also induced in activated CD4^+^ effector T cells (Teffs). This study aims to leverage adult peripheral blood, umbilical cord blood, and pediatric thymic tissue to precisely characterize human Tregs and to gain deeper insights into heterogeneity across sources and developmental stages.

**Methods:**

We conducted extensive flow cytometric analysis of 31 extra- and intracellular markers expressed by human Tregs, followed by an in-depth comparison of Tregs and Teffs, as well as between Tregs derived from all three sources.

**Results:**

Our results showed that, while most markers were shared with Teffs, the transcription factor Helios, the co-inhibitory receptors CTLA-4 and TIGIT, and the glycoprotein receptor GPA33 were expressed by a higher proportion of Tregs than Teffs across sources. Contrary, a consistently higher proportion of Teffs than Tregs expressed the co-stimulatory receptors CD26 and CD226. Thymocytes displayed marked heterogeneity, containing Tregs at distinct developmental stages and recirculating peripheral Tregs. The proportion of CD25^^+^^FOXP3^lo/–^ CD4 single positive (SP) precursor cells expressing Treg specific markers Helios, TIGIT and CTLA-4 was significantly lower than of CD25^+^FOXP3^^+^^ double-positive (CD4^+^CD8^+^) Tregs and mature CD25^+^FOXP3^+^ CD4SP Tregs. These findings challenge the previously held notion that CD25^+^FOXP3^lo/–^ precursors uniformly mature into CD25^+^FOXP3^+/hi^ Thy-Tregs. As this subset differs from mature Thy-Tregs primarily by FOXP3 expression - a marker unsuitable for live-cell sorting - conventional isolation methods fail to exclude this immature subset. Importantly, our study identified the surface markers CD45RA/CD45RO, GPA33, TIGIT, and PD-1 to exclusively distinguish mature Thy-Tregs from these precursors. Moreover, our study provides a detailed characterization of highly activated recirculating peripheral Tregs within the thymocytes. Among the three sources examined, cord blood-derived Tregs exhibited the greatest phenotypic uniformity compared with adult blood- and thymus-derived Tregs.

**Conclusion:**

Overall, this study provides highly detailed insights into the heterogeneity of Tregs across distinct developmental stages and therapeutic sources, while also contributing towards improved isolation strategies for therapeutic approaches.

## Introduction

FOXP3^+^ regulatory T-cells (Tregs), comprising 4-7% of the CD4^+^ helper T-cell population, have been the focus of intense research due to their critical role in maintaining immune homeostasis and inducing peripheral tolerance. Early phase clinical trials have demonstrated proof-of-principle for the use of isolated and expanded polyclonal Tregs in the treatment of inflammatory disorders, transplant rejection and autoimmune diseases (1). However, despite the appealing prospects of Treg-based therapies, the ongoing and completed Treg clinical trials have shown varying *in vivo* persistence and clinical efficacy (2). This variability is presumably linked to both known and unknown differences in the sources of Tregs, different *ex vivo* expansion processes and resulting phenotype of the infused products, highlighting the need for a deeper understanding of Treg heterogeneity across different sources.

Currently, CD3^+^CD4^+^CD8^-^CD25^+^CD127^lo^ remains the most widely used gating strategy for the isolation of viable Tregs by fluorescence activated cell sorting (FACS), with subsequent intranuclear staining of FOXP3 to confirm purity and function. Briefly, CD25, the IL-2 receptor α-chain, is a canonical marker for Tregs (3, 4). CD127, the IL-7 receptor α-chain, is known to inversely correlate with FOXP3, a forkhead box transcription factor, that serves as the master regulator for Treg development and function (5, 6). However, the precise identification of human Tregs is challenging, because the signature proteins are mostly shared by activated CD4^+^ effector T cells (Teffs) (7). Of note, FOXP3, whilst exclusive to Tregs in mice, does not provide unambiguous identification of human Tregs (8). Thus, finding specific markers for human Tregs remains an unmet need. Over the past two decades, numerous novel markers associated with Treg origin, maturity, stability, and function have been identified (9). Most of these studies, however, have primarily focused on adult peripheral blood-derived mononuclear cells (PBMCs) as Treg source (10, 11). Due to the increasing therapeutic interest in umbilical cord blood mononuclear cells (CBMCs) (12–14) and discarded pediatric thymus-derived Tregs (15–17), there is a growing need for a comprehensive comparison of Treg populations within these sources. Hence, in-depth phenotypic analysis of all three source materials will help identify markers universally specific to Tregs or Teffs, facilitating a better understanding of contaminating Teffs in the infused product and Treg heterogeneity across these sources.

Current understanding of Treg development largely stems from mouse studies. However, technical limitations and the lack of specific tools have hindered a full definition of human Treg development. Nevertheless, several insights into human thymic Treg development have been reported (18–20). Tregs primarily develop in the thymus (natural Tregs, nTregs). Current evidence suggests that Treg lineage commitment in humans may occur at multiple stages of T cell development (18, 20). The most immature thymocyte population in humans that clearly expresses FOXP3 and displays regulatory function is the cortical CD4^+^CD8^+^ double positive (DP) subset (21–23). The FOXP3^+^ DP cells strongly correlate with the FOXP3^+^ CD4 single positive (SP) population, suggesting a precursor–product relationship (23). Medullary CD4SP FOXP3^lo/-^ thymocytes can receive TCR and costimulatory signals that induce CD25 expression, giving rise to Treg precursors (CD4SP CD25^+^FOXP3^lo/-^). These precursors can then differentiate into thymic Tregs in response to IL-2 or IL-15 (24, 25). Outside the thymus, Tregs can also be differentiated in the periphery from CD4^+^ Teffs (peripherally-induced Tregs, pTregs) (26, 27). The distinction between nTregs and pTregs within the human peripheral Treg compartment is challenging due to the lack of specific markers. Recent studies have also shown that the circulating peripheral CD25^+^FOXP3^+/hi^ Tregs have the ability to migrate back into the thymus (28–30). Thus, the thymus contains a heterogeneous Treg population comprising both developing and recirculating cells. With thymus-derived Tregs moving toward clinical translation, detailed phenotyping of developing and recirculating Tregs in the human thymus—and a deeper understanding of their heterogeneity—is essential.

In this study, we explored naturally occurring therapeutic sources—PBMCs, CBMCs, and thymocytes—to precisely characterize human Tregs and gain deeper insights into their heterogeneity across sources and developmental stages. We performed an extensive flow cytometric analysis of 31 published markers present in human Tregs (9) to gain a better understanding of: 1) the phenotypic differences between Teffs and Tregs, compared across PBMCs, CBMCs, and thymocytes; 2) the phenotype of Tregs across developmental stages, including the identification of recirculating peripheral Tregs within the thymocytes; and 3) the heterogeneity of Tregs across PBMCs, CBMCs, and thymocytes. Overall, this study aims to provide a comprehensive definition of the human Treg compartment and to identify phenotypic markers with potential utility for optimizing Treg isolation in therapeutic settings.

## Materials and methods

The minimum information about Treg cells (MITREG) checklist was followed for the preparation of this paper (31). See http://w3id.org/ontolink/mitreg for MITREG document and checklist.

### Blood samples

Buffy coat preparations from adult peripheral blood were obtained from the German Red Cross (Deutsches Rote Kreuz, DRK; Blutspendedienst Nord-Ost GmbH Dresden) released for research under informed consent. Fresh umbilical cord blood donations, collected from full-term neonates and processed within 24 hours of birth, were sourced from the DKMS (German Bone Marrow Registry) Stem Cell Bank in Dresden with ethics committee approval (approval number: EK 363102011). These cord blood samples were released for research under informed consent, due to insufficient cell count or volume to meet the Cord Blood Bank´s criteria for clinical use. Cord blood donations were received in clinical grade collection bags containing 35 mL of citrate phosphate dextrose anticoagulant (Macopharma).

### Isolation of blood mononuclear cells

Mononuclear cells were isolated from human peripheral blood (PBMCs) and cord blood (CBMCs). Adult buffy coat or whole cord blood was first diluted 1:3 with sterile PBS. Thirty milliliters of the diluted blood were carefully layered on top of 15 ml of Ficoll lymphocyte separation medium (Lymphoprep; Axis Shield) in a 50 ml conical tube and centrifuged at 650 g for 25 min at room temperature (RT) with the centrifuge brake switched off. Following centrifugation, mononuclear cells were recovered as white layer at the interface between the Ficoll reagent and the Plasma/PBS using a plastic Pasteur pipette, then washed once with 45 ml of sterile PBS by centrifugation at 300 g for 15 min at RT with medium braking. Thrombocytes were removed in a subsequent washing step at 200 g for 15 min at RT. Cells were suspended in RPMI complete medium, consisting of RPMI supplemented with 10% human AB serum (PAN Biotech), 1% sodium pyruvate (Sigma-Aldrich), 1% non-essential amino acids (NEAA; Sigma-Aldrich), and 1% glutamate (Sigma-Aldrich). Cells were incubated at 37°C with 5% CO_2_ for up to 16 hours before further processing.

### Thymic tissue

Thymic tissue from the pediatric patients (three males, one female, aged between 2 to 12 months) were collected during routine cardiac surgeries at Pediatric Cardiac Surgery Unit, Heart Center Leipzig, Germany. The excised thymic tissue, which would otherwise be discarded, were transported in sterile containers with NaCl supplemented with 1% antibacterial and antifungal antibiotic (Penicillin-Streptomycin-Amphotericin B; Sigma-Aldrich). The samples were maintained at 4°C and processed within 5-7h post-surgery. This study was conducted with approval from the ethics committee (approval number: SR + BO-EK-323072023), with written informed consent obtained from the legal guardians before surgery.

### Isolation of thymocytes

The obtained thymus tissue (10–20 g) was thoroughly washed in sterile PBS, then cut into pieces of approximately 1–2 g using surgical scissors. These pieces were placed into a 5 ml Falcon tube with 1 ml of digestion medium: TexMACS medium (Miltenyi Biotec) containing 1 µl of Benzonase^®^ Nuclease (EMD Millipore) per 500 µl of medium. The tissue was further cut into 2–3 mm fragments inside the Falcon tube using smaller surgical scissors, and mechanically disaggregated for 40 minutes using the gentleMACS Dissociator (Miltenyi Biotec) with a customized protocol for human thymus tissue digestion. The digested fraction was serially filtered through 100 µm followed by 40 µm pore cell strainers to remove undigested clumps and aggregates, resulting in a single-cell suspension of thymocytes. The thymocytes were washed twice with 45 ml of sterile PBS by centrifugation at 300 g for 15 min at RT. Cells were suspended in RPMI complete medium, consisting of RPMI supplemented with 10% human AB serum (PAN Biotech), 1% sodium pyruvate (Sigma-Aldrich), 1% non-essential amino acids (NEAA; Sigma-Aldrich), and 1% glutamate (Sigma-Aldrich). Cells were incubated at 37°C with 5% CO_2_ for up to 16 hours before further processing.

### Cell counting

For cell counting, a 10 µl aliquot of the cell suspension was stained with 3 µl of 7-AAD Staining Solution (Miltenyi Biotec) and 1 µl of anti-human CD45 antibody conjugated with VioBlue (Miltenyi Biotec) for 15 min at RT. After adequate dilution, the samples were measured using the MACSQuant^®^ Analyzer 10 (Miltenyi Biotec). 7-AAD positive staining was used to determine the dead cell content, while CD45 staining was used to exclude CD45^neg^ nucleated red blood cells (NRBCs) in CBMCs and other non-hematopoietic cells from thymocytes.

### Cell staining and phenotypic analysis

Based on previous studies, which we recently summarized (9), we assessed the phenotype of Tregs and Teffs within PBMCs, CBMCs and thymocytes using 31 markers (4 intracellular and 27 extracellular) alongside standard Treg markers (CD3, CD4, CD8, CD25, CD127, FOXP3, and a fixable viability dye). All antibodies used are listed in **Supplementary Table 1**. Panel composition is provided in **Supplementary Table 2**.

For staining of cell surface markers, 1-2 × 10^6^ cells were washed with 1 ml of FACS buffer (autoMACS Rinsing Solution (Miltenyi Biotec) + 5% MACS BSA Stock Solution (Miltenyi Biotec)) by centrifugation at 300 g for 10 min at 4°C. The supernatant was discarded, and 5 µl of FcR Blocking Reagent (Miltenyi Biotec), diluted with 45 µl of PBS, was added to the cell pellet. Cells were incubated on ice for 10 min. Subsequently, 50 µl of Brilliant Stain Buffer (BD Biosciences) and 1-10 µl of fluorochrome-conjugated antibodies were added to the cell suspension. The titrated antibody volumes utilized in this study are outlined in **Supplementary Table 1**. After staining on ice for 25–30 min, cells were washed twice with 1 ml of FACS buffer by centrifugation at 300 g for 10 min at 4°C.

For staining of transcription factors such as FOXP3, Helios, and other intracellular markers like CTLA-4 and TGF-β, the eBioscience Foxp3/Transcription Factor Staining Buffer Set (Invitrogen, Thermo Fisher Scientific) was used. Briefly, extracellular marker-stained cells were resuspended in 1 ml of Fix/Perm solution prepared according to the manufacturer´s recommendation, vortexed, and incubated at RT in the dark for 45 min. Cells were then centrifuged and washed twice with 2 ml of 1X working solution of Permeabilization Buffer by centrifugation at 500 g for 10 min at RT. The supernatant was discarded and cells were stained with 2-4 µl of fluorochrome-conjugated intracellular antibodies for 25–30 min at RT in the dark. Cells were subsequently washed twice with 1 ml of 1X Permeabilization Buffer by centrifugation at 500 g for 10 min at RT. Finally, the fixed and stained cells were resuspended in 150-200 µl of FACS buffer and kept in the fridge for up to 48 h until flow cytometric analysis.

Single-color compensation controls were prepared using anti-mouse and anti-rat Igκ-coated polystyrene beads, as well as anti-REA beads (MACS Comp Bead Kits, Miltenyi Biotec) according to the manufacturer´s recommendations. To prepare compensation controls for the viability dye, freshly isolated cells were heat-killed at 65°C for 10 min and then placed on ice for an additional 10 min. The heat-killed cells were then mixed with an equal volume of live cells and stained with the viability dye. Cells were subsequently fixed using the previously described fixation protocol.

Phenotypic analysis was conducted using a BD LSR Fortessa flow cytometer equipped with 5 lasers (355 nm, 405 nm, 488 nm, 561 nm, 640 nm). Compensation was performed using the automatic compensation tool in BD FACSDiva software. Samples were recorded at 500–2500 events per second. A total of 50,000 events within the live CD3^+^CD4^+^ cell gate was acquired. To standardize the flow cytometric readouts across time, same application settings were used in each experiment.

### Data analysis

Flow cytometry data were analyzed using FlowJo v10.10.0 software (FlowJo LLC, Ashland, OR, USA). The gate for positive cells was set using internal negative controls (INCs), cells in the staining sample that do not express the marker of interest. Since most markers are specific to the T cell lineage (CD3^+^), non-T cells (CD3^-^CD4^-^) were used as INCs in our experiments. GraphPad PRISM v10.3.1 (GraphPad Software Inc., California, USA) was used for data visualization and statistical analysis. Normality testing was performed in Microsoft Excel. Unless otherwise stated, the data followed a non-Gaussian distribution and are presented as medians with interquartile range. We applied the unpaired t-test with Welch’s correction for comparing two independent groups or Kruskal–Wallis test followed by the Dunn *post hoc* test for multiple comparisons. P values: *P < 0.05, **P < 0.01, ***P < 0.001, ****P < 0.0001.

## Results

### Marked T cell heterogeneity characterizes thymocytes compared with cord blood and adult peripheral blood

**Figure 1:**
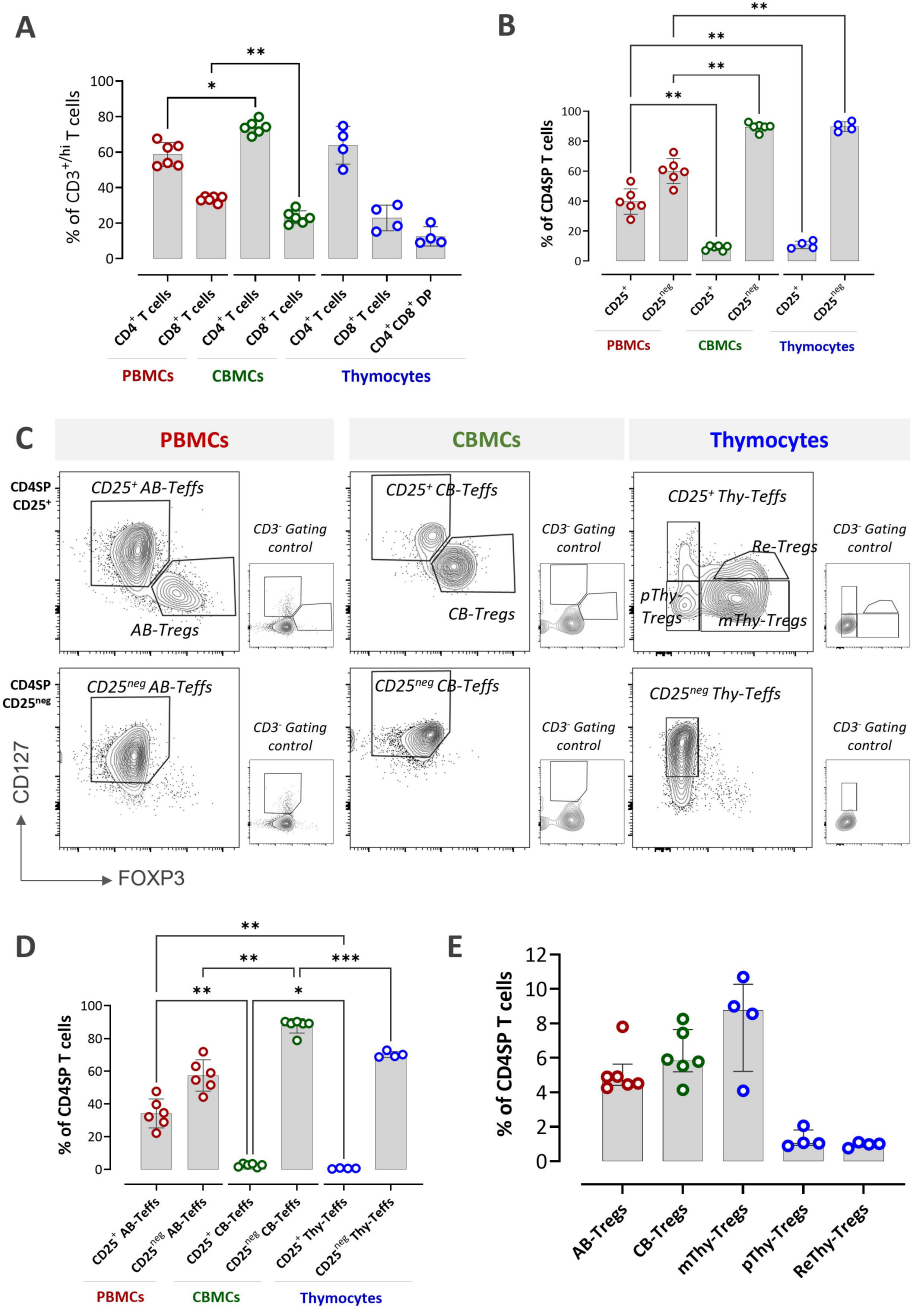
Composition of Teff and Treg subsets within PBMCs, CBMCs, and thymocytes. **(A)** The percentage of CD4^+^, CD8^+^, and CD4^+^CD8^+^ DP T cells within CD3^+/hi^ T cells. **(B)** The percentage of CD25^+^ and CD25^neg^ populations within CD4SP T cells. **(C)** Representative flow plots illustrating Teff and Treg subsets within CD4SPCD25^+^ and CD4SPCD25^neg^ cells. CD3^-^ gating controls are shown in smaller plots adjacent to each corresponding plot. **(D)** The percentage of CD25^+^ Teffs and CD25^neg^ Teffs within CD4SP T cells. **(E)** The percentage of Treg subsets within CD4SP T cells. Data are shown as bar plots with median and interquartile range [PBMCs (red)/CBMCs (green): n=6; thymocytes (blue): n=4]. Statistical testing was performed using the Kruskal–Wallis test with Dunn’s *post hoc* test and Holm adjustment for multiple comparisons (two-sided, α = 0.05) (*P<0.05, **P<0.01, ***P<0.001). AB, adult blood; CB, cord blood; Thy, thymus; DP, double positive; SP, single positive; pThy-Tregs, precursor thymic Tregs; mThy-Tregs, mature thymic Tregs; Re-Tregs, recirculating peripheral Tregs.

Different T cell populations within freshly isolated PBMCs, CBMCs, and thymocytes were identified using manual gating strategies (**Supplementary Figures 1**–**3**). In PBMCs and CBMCs, a clearly defined CD3^+^ population was detected; however, thymocytes displayed three subsets based on CD3 expression: CD3^neg/lo^, CD3^med^, and CD3^hi^. In previous literature, CD3^+^CD8^-^CD25^+^ cells were used as the gating strategy before analyzing FOXP3 expression in thymocytes (32). In our study, when we examined the CD25 expression of CD3^neg/lo^ and CD3^med^, and none of these populations expressed CD25 (**Supplementary Figure 3**). Hence, the CD3^hi^ subset was considered representing the most mature T cells, was selected for further analysis. Across all three sources, CD4^+^ T cells constituted over half of the CD3^+/hi^ T cell population. This proportion was highest in CBMCs (median 73.9%; range 68.7–79.8%), compared with PBMCs (median 58.7%; range 52.5–67.6%) and thymocytes (median 63.8%; range 50.1–74.7%) (**Figure 1A**). Notably, a CD4^+^CD8^+^ double-positive (DP) subset was detected within CD3^hi^ thymocytes, with a median frequency of 12.53% (range 8.9–20.5%). CD4^+^ T cells were further characterized based on CD25 expression. In general, CD25^neg^ cells were more prevalent than CD25^+^ cells across all sources; however, the highest proportion of CD25^+^ cells were observed in PBMCs (**Figure 1B**).

Teffs were defined as CD127^+/hi^FOXP3^lo^ within both CD25^+^ and CD25^neg^ CD4^+^ subsets, referred to as CD25^+^ Teffs and CD25^neg^ Teffs, respectively (**Figure 1C**). Because CD25 is a marker of T-cell activation, its expression allowed discrimination between antigen-experienced Teffs (CD25^+^) and naïve or less activated Teffs (CD25^neg^). The proportion of CD25^neg^ Teffs within CD4 single-positive (CD4SP) cells was significantly higher in CBMCs (median 87.8%, range 78.8–90.3%) compared with PBMCs (median 57.4%, range 22.2–47.7%) and Thymocytes (median 70.2%, range 68.7–72.8%) (**Figure 1D**). In contrast, activated CD25^+^ Teffs were rare in thymocytes (<1%) and CBMCs (0.8–2.5%), but were most abundant in PBMCs (median 35%, range 20–46.9%).

Tregs were defined as CD127^lo^FOXP3^+/hi^ within CD25^+^ compartment (**Figure 1C**). In line with previous studies (33–35), Treg frequencies within CD4SP cells were comparable in PBMCs and CBMCs, with medians of 4.9% (range 4.3–7.8%) and 5.9% (range 3.9–8%), respectively. In contrast, thymocytes exhibited a relatively higher frequency of CD127^lo^FOXP3^+/hi^ mature thymic Tregs (mTregs) without reaching statistical significance (median 8.5%, range 4–10.7%) (**Figure 1E**). Within CD25^+^CD4SP Thymocytes, alongside mThy-Tregs, two additional subsets were identified: a CD127^lo^FOXP3^lo^ population (median 1.05%, range 0.89–2.06%) corresponding to precursor thymic Tregs (pThy-Tregs) as previously described (32), and a CD127^med^FOXP3^hi^ subset (median 1.0%, range 0.76–1.11%) hypothesized to represent recirculating peripheral Tregs (Re-Tregs), in line with earlier observations (29). Taken together, the presence of multiple Treg subsets at distinct developmental stages makes the thymocytes the most heterogeneous source examined for Tregs.

### Tregs and Teffs exhibit the most distinct phenotypic profiles in umbilical cord blood

**Figure 2:**
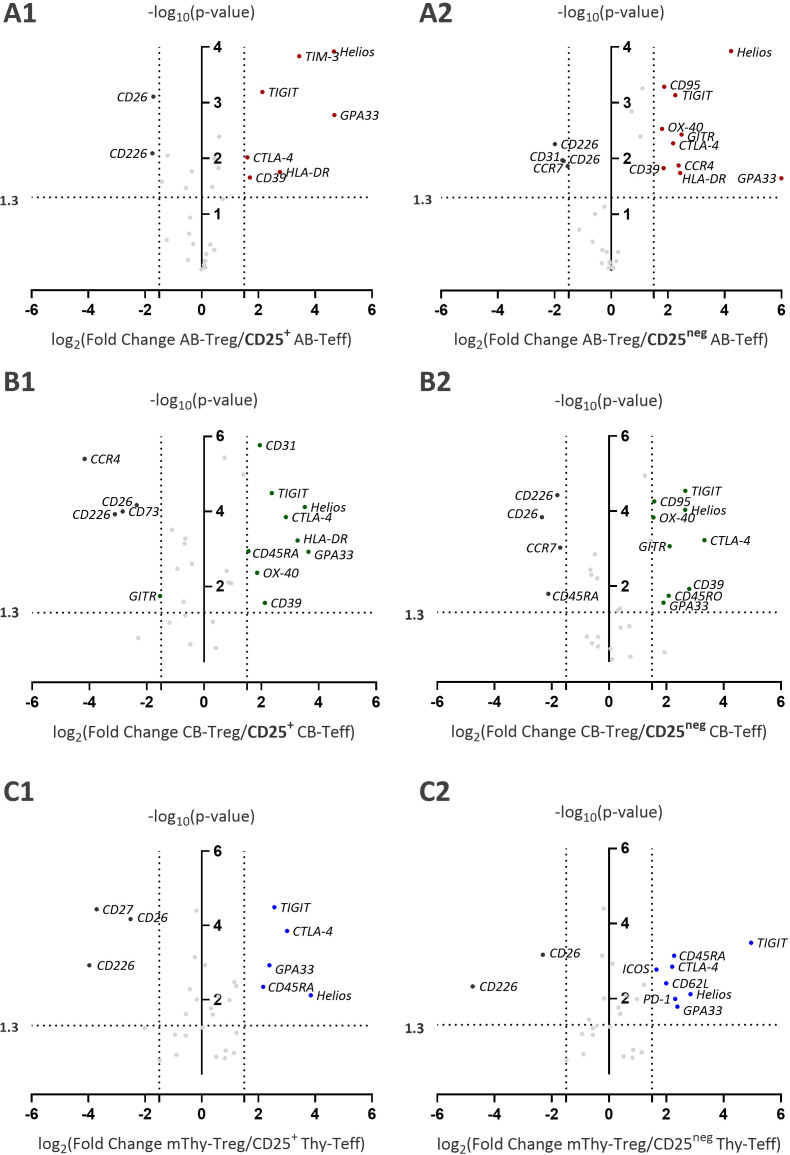
Phenotypic differences between Treg and Teff subsets across PBMCs, CBMCs, and thymocytes. A log_2_ fold-change versus –log_10_ p-value (volcano) plot was used to compare expression levels of 31 markers between Tregs and Teffs and to assess statistical significance. (A1) AB-Tregs vs CD25^+^ AB-Teffs (A2) AB-Tregs vs CD25^neg^ AB-Teffs (B1) CB-Tregs vs CD25^+^ CB-Teffs (B2) CB-Tregs vs CD25^neg^ CB-Teffs (C1) mThy-Tregs vs CD25^+^ Thy-Teffs (C2) mThy-Tregs vs CD25^neg^ Thy-Teffs. The x-axis shows the log_2_ fold change in mean fluorescence intensity (MFI) (Treg/Teff ratio), where positive values indicate higher expression in Tregs and negative values indicate higher expression in Teffs. A fold-change threshold of ±1.5 was applied. The y-axis (–log_10_ p-value) represents statistical significance, with values >1.3 considered significant (p<0.05; Mann–Whitney U test). Median MFI values from independent donors [PBMCs and CBMCs (n=5) and thymocytes (n=4)] were used for this analysis. AB, adult blood; CB, cord blood; Thy, thymus; mThy-Tregs, mature thymic Tregs.

**Figure 3:**
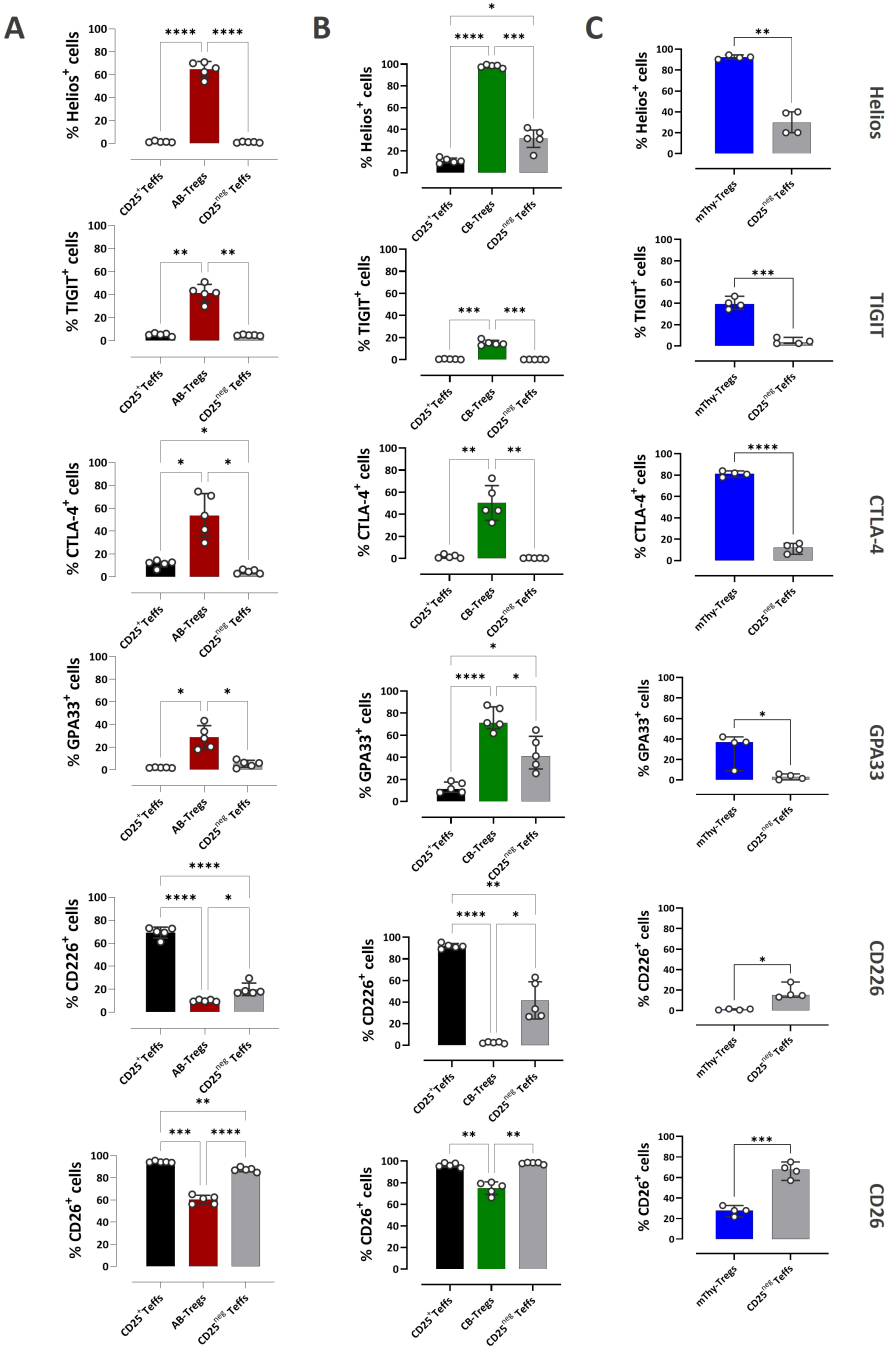
Phenotypic differences between Treg and Teff subsets across PBMCs, CBMCs, and thymocytes. Bar graphs showing the percentage of Treg and Teff subsets within **(A)** PBMCs, **(B)** CBMCs, and **(C)** thymocytes expressing selected markers identified from the comparative phenotypic analysis. Data are presented as bar plots with median and interquartile range [PBMCs (red) and CBMCs (green): n=5; thymocytes (blue): n=4]. Statistical testing was performed using unpaired t-test with Welch’s correction for comparing two independent groups and using Kruskal–Wallis test with Dunn’s *post hoc* test and Holm adjustment for multiple comparisons (two-sided, α = 0.05) (*P<0.05, **P<0.01, ***P<0.001, ****P<0.0001). AB, adult blood; CB, cord blood; Thy, thymus; mThy-Tregs, mature thymic Tregs.

Of the 31 analyzed markers, most were expressed by both Tregs and Teffs across all three sources (**Supplementary Figures 4**–**6**). Hence, a comparative analysis was performed to assess differences in expression levels between Tregs and Teffs. Furthermore, because CD25 expression within the CD4^+^ T-cell compartment follows a continuum rather than a binary pattern, Tregs were compared with both CD25^+^ Teffs and CD25^neg^ Teffs. To achieve this, we plotted the log_2_ fold change in MFI of Tregs versus Teffs for the tested markers against the -log_10_ p-value. To minimize fold-change inflation, a baseline threshold corresponding to 1.5× the MFI fold change was applied. The extent of phenotypic separation between Tregs and Teffs differed by source. In PBMCs, significant expression differences were observed for 14 markers between Tregs and CD25^neg^ Teffs, and for 9 markers between Tregs and CD25^+^ Teffs, proving that CD25^neg^ Teffs were more phenotypically distinct from Tregs (**Figures 2A1**, **A2**). In CBMCs, 14 markers showed significantly different expression between Tregs and CD25^+^ Teffs, and 13 markers differed in expression between Tregs and CD25^neg^ Teffs (**Figures 2B1, B2**). Thymocytes showed the least distinction, with only 8 and 10 markers differing between Tregs and CD25^+^ and CD25^neg^ Teffs respectively (**Figures 2C1**, **C2**). Despite this variability, a conserved set of markers consistently showed higher level of expressions in Tregs than in Teffs across all three sources and regardless of CD25 status. These included the surface markers TIGIT (36, 37) and GPA33 (38, 39), and intracellular expression of the co-inhibitory receptor CTLA-4 (40) and the transcription factor Helios (9, 41). Conversely, the co-stimulatory molecules CD226 (42) and CD26 (43) remained highly expressed in both CD25^+^ and CD25^neg^ Teffs across all tissues examined. These six markers were selected for further comparison and the percentage expression of Tregs and Teffs were plotted (**Figure 3**).

Because CD25^+^ Teffs represented <1% of CD4SP thymocytes, this subset was excluded from the analysis. Across all three sources, a significantly higher proportion of Tregs expressed Helios, TIGIT, CTLA-4, and GPA33 compared with both CD25^+^ and CD25^neg^ Teffs (**Figures 3A–C**). In most cases, fewer than 5% of Teffs expressed these markers, with a few notable exceptions mainly in case of CD25^neg^ Teffs. For example, 31.5% of CD25^neg^ Teffs in CBMCs (range 15.8–41.5%) and 29.6% in thymocytes (range 20.1–40%) expressed Helios. Similarly, CTLA-4 expression was detected in 12.4% of CD25^+^ Teffs in PBMCs (range 6.17–14.50%) and 12.1% of CD25^neg^ Teffs in thymocytes (range 5.88–16.1%). A higher proportion of CD25^neg^ Teffs in CBMCs expressed GPA33, with a median of 41.2% (range 25.4–64.7%). In contrast, a significantly higher proportion of Teffs expressed CD226, whereas fewer than 10% of Tregs across all these three sources expressed this marker (**Figures 3A–C**). Although CD26 expression was also lower in Tregs compared with Teffs, a substantial proportion of Tregs still expressed CD26, with median values exceeding 50% in both PBMCs and CBMCs.

Moreover, we compared the expression of these markers in ThyTregs at different developmental stages—including DP Tregs, pThy-Tregs, and mThy-Tregs. CTLA-4, TIGIT, and Helios expression was significantly higher in all three Treg populations than in CD25^neg^ Teffs (**Supplementary Figure 7**). In contrast, expression of the co-stimulatory receptors CD26 and CD226 was lower across all Treg subsets compared with CD25^neg^ Teffs. Together, these findings suggest that these markers may be associated with the commitment to the Treg and Teff developmental lineages in the thymus.

### Distinct Treg marker profiles across adult peripheral blood, cord blood and thymocytes

**Figure 4:**
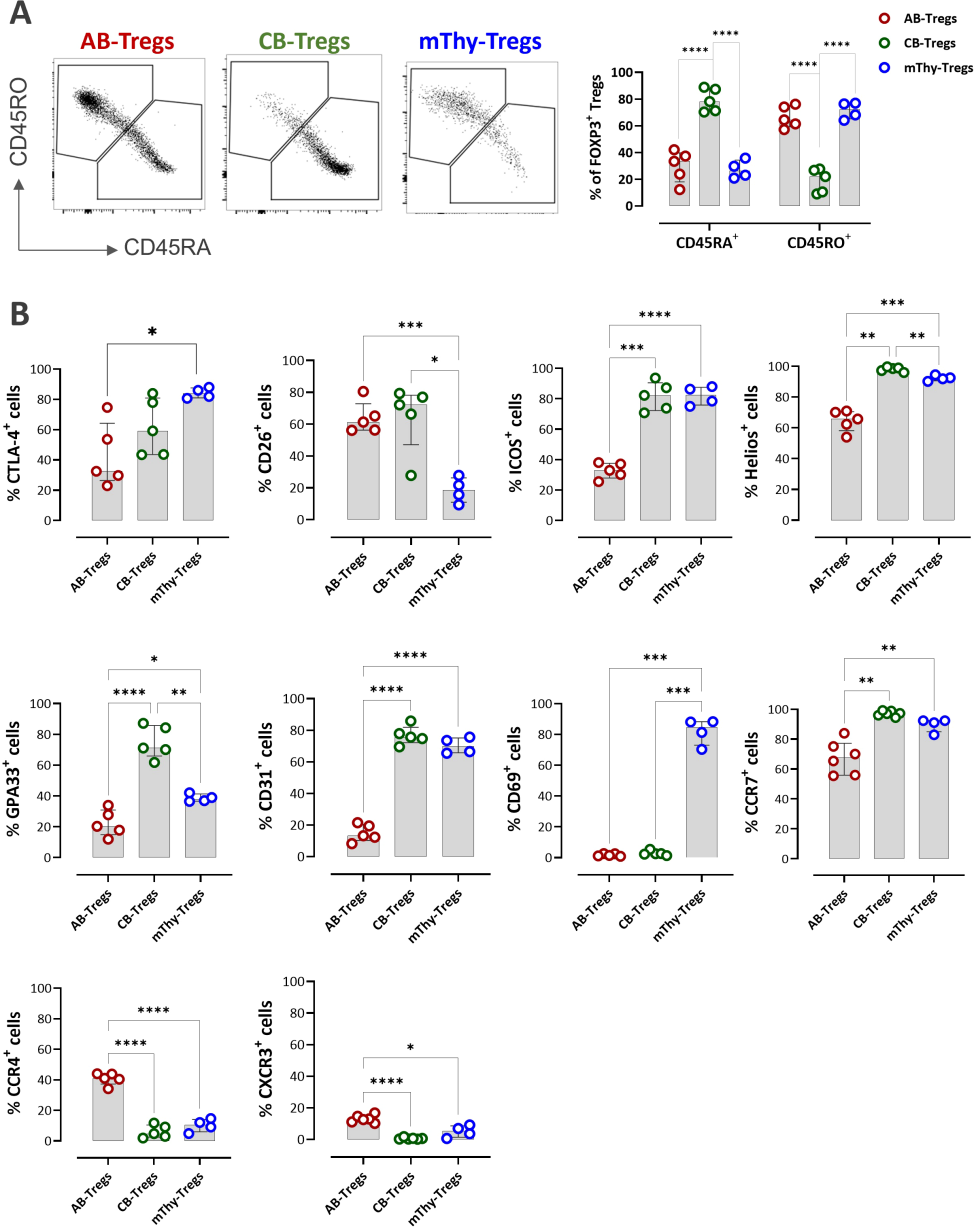
Phenotypic heterogeneity of CD127^lo^FOXP3^+/hi^ Tregs within PBMCs, CBMCs, and thymocytes. (**A**) Representative dot plots and bar graph showing the distribution and percentage of CD45RA^+^ naïve and CD45RO^+^ memory populations within CD127^lo^FOXP3^+/hi^ Tregs respectively. **(B)** Bar graphs showing the percentage of AB-Tregs, CB-Tregs, and mThy-Tregs expressing certain selected markers. Data are presented as bar plots with median and interquartile range [PBMCs (red)/CBMCs (green): n=5; thymocytes (blue): n=4]. Statistical testing was performed using the Kruskal–Wallis test with Dunn’s *post hoc* test and Holm adjustment for multiple comparisons (two-sided, α = 0.05) (*P<0.05, **P<0.01, ***P<0.001, ****P<0.0001). AB, adult blood; CB, cord blood; Thy, thymus; mThy-Tregs, mature thymic Tregs.

Next we performed comparative analysis of marker expression profiles within Treg population. It revealed source-specific Treg signatures and phenotypic heterogeneity among AB-Tregs, CB-Tregs and mThy-Tregs. Consistent with previous studies (44), 37.4% of AB-Tregs expressed CD45RA (range 12.3–42.1%), whereas 64.5% expressed CD45RO (range 57.5–76.2%), indicating the predominance of a memory phenotype and greater antigen experience (**Figure 4A**), known to correlate with donor age (45). In contrast, mThy-Tregs derived from thymus donors aged 2–12 months, although expected to exhibit a largely naïve phenotype, only 23.0% (range 20.9–35.9%) of them expressed CD45RA, reflecting their transitional developmental state (46). Among the three sources, CB-Tregs displayed the highest proportion of CD45RA^+^ cells, with a median of 78.1% (range 65.2–79.6%).

The expression of co-stimulatory and co-inhibitory receptors on Tregs varied across sources without a consistent pattern (**Supplementary Figures 8A, B**). Across all three sources, expression of the co-stimulatory receptors CD137 and OX-40, as well as the co-inhibitory receptors PD-1, LAG-3, and TIM-3, remained low, likely reflecting the freshly isolated and unstimulated state of the cells (**Supplementary Figures 8A, B**). Notably, a higher percentage of mThy-Tregs exhibited CTLA-4 expression and a lower percentage expressed CD26 compared with AB-Tregs and CB-Tregs (**Figure 4B**). CB-Tregs displayed a lower TIGIT^+^ fraction compared with AB- and mThy-Tregs; however, the difference was not statistically significant. Additionally, a lower percentage of AB-Tregs expressed ICOS, suggesting reduced functional quality, as this marker is associated with enhanced and suppressive capacity (47).

The proportion of CB-Tregs expressing Helios, GPA33, and the recent thymic emigrant marker CD31—previously reported to be associated with Treg quality and lineage stability (9)— was significantly higher than in AB-Tregs and mThy-Tregs (**Figure 4B**). Notably, more than 80% of mThy-Tregs expressed CD69, an early activation marker, compared with only 1–2% of AB-Tregs and CB-Tregs, suggesting ongoing self-antigen exposure within the thymic environment. In addition, most CB-Tregs did not express the chemokine receptors CCR4 or CXCR3 but did express the lymphoid-homing receptor CCR7, consistent with their naïve phenotype and supporting the overall homogeneity of this subset.

### Additional surface markers enable the isolation of pure mature thymic Tregs

**Figure 5:**
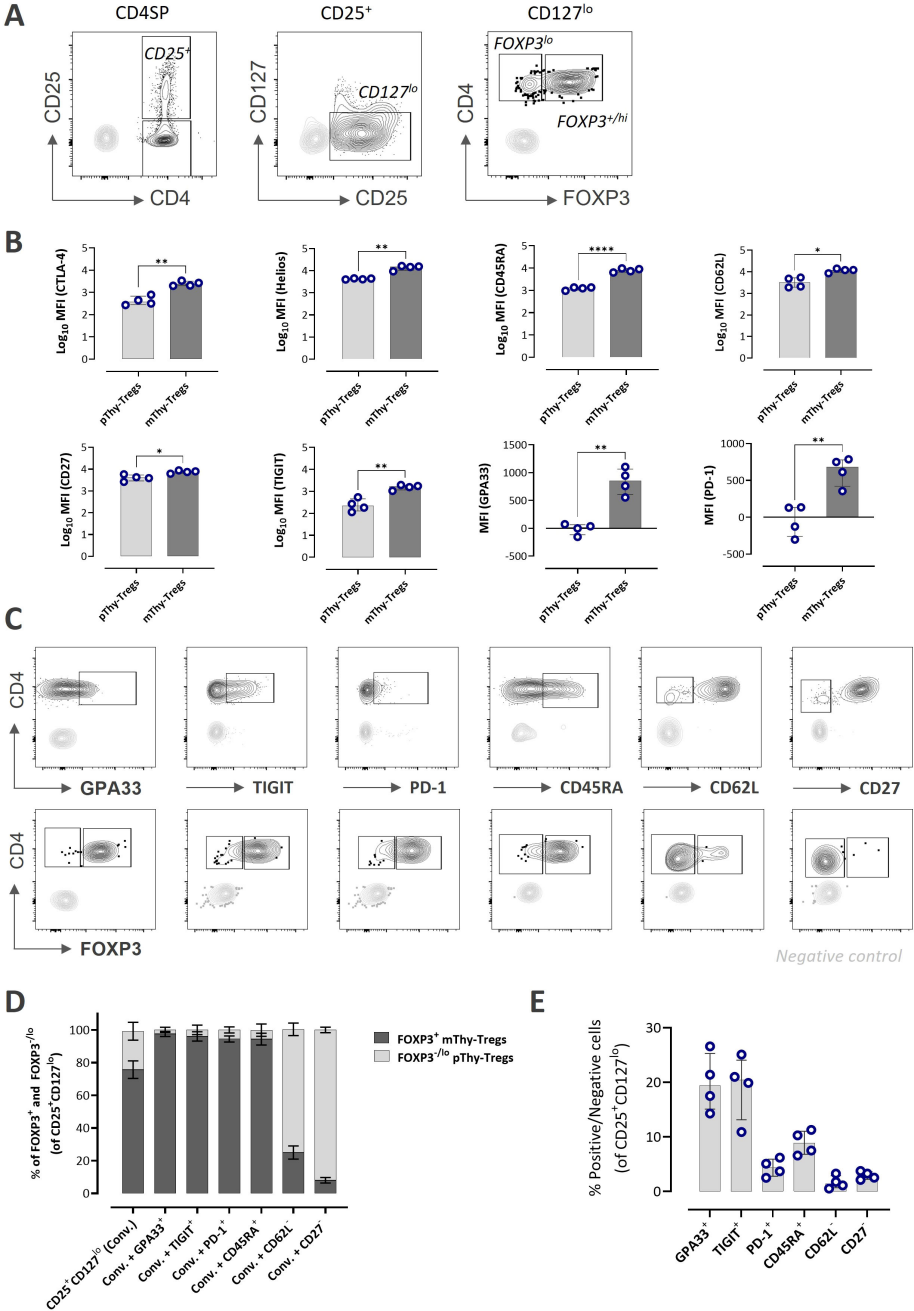
Phenotypic differences between precursor and mature thymic Tregs. **(A)** Representative conventional Treg gating strategy of CD25^+^CD127^lo^ to isolate viable bulk Tregs, along with the distribution of FOXP3^+/hi^ and FOXP3^lo^ cells within that population. CD3^-^ gating control populations are overlayed in grey in each corresponding plots. **(B)** Bar graphs showing the log_10_- transformed mean fluorescence intensity (MFI) values of selected markers within pThy-Tregs and mThy-Tregs. GPA33 and PD-1 contain data points with negative MFI values and were therefore presented as absolute values. **(C)** Representative flow plots (top) showing selected marker-defined populations (GPA33^+^, TIGIT^+^, PD-1^+^, CD45RA^+^, CD62L^-^, CD27^-^) within conventional CD3^hi^CD4SPCD25^+^CD127^lo^ Thy-Tregs, and their corresponding FOXP3^+/hi^ and FOXP3^lo^ distributions (bottom). CD3^-^ gating control populations are overlayed in grey in each corresponding plots. **(D)** Stacked bar graph showing the proportions of FOXP3^+^/^hi^ (dark grey) and FOXP3^-^/^lo^ (light grey) cells within each marker-defined subset. **(E)** Bar graph summarizing the frequencies of selected populations within conventional Thy-Tregs. Data are presented as median with interquartile range (n=4). Statistical analysis was performed using unpaired t-test with Welch’s correction for comparing two independent groups (*P<0.05, **P<0.01, ****P<0.0001). SP, single positive; Thy, thymus; pThy-Tregs, precursor thymic Tregs; mThy-Tregs, mature thymic Tregs.

When we applied the conventional sorting gating strategy of CD4^+^CD8^-^CD25^+^CD127^lo^ to thymocytes, we identified two distinct Treg subsets: a FOXP3^lo^ pThy-Tregs and a FOXP3^+^/^hi^ mature population (mThy-Tregs) (**Figure 5A**). We compared the expression level (MFI) of 31 markers between these two population, most markers showed no significant differences (**Supplementary Figure 6A**), with only a few notable exceptions: Expression of the co-stimulatory receptor CD27, the co-inhibitory receptors CTLA-4 (intracellular), TIGIT, and PD-1, Treg-associated markers such as Helios and GPA33, and naïve markers including CD45RA and CD62L were significantly lower in pThy-Tregs compared with mThy-Tregs (**Figure 5B**). Most of these markers are reported to be associated with Treg quality and functionality (9), and the differentiation and phenotype after *in vitro* expansion of pThy-Tregs are not yet completely understood. Therefore, for therapeutic applications, it may be important to remove these precursor cells. However, because pThy-Tregs differ from mThy-Tregs only based on FOXP3 expression (**Figure 5A**)—and FOXP3 is an intranuclear marker unsuitable for live-cell sorting—conventional isolation strategies are unable to reliably exclude these cells. Therefore, we conducted further analyses to determine whether incorporating specific surface markers would enable the separation of FOXP3^lo/-^ p-Thy-Tregs from FOXP3^+/hi^ mThy-Tregs.

Most of the markers analyzed showed overlapping expression patterns between pThy-Tregs and mThy-Tregs, except for six surface markers that showed potential to distinguish the two populations (**Supplementary Figure 9**): GPA33, CD45RA/RO, PD1 and TIGIT for sorting FOXP3^+/hi^ cells, while CD27 and CD62L for excluding FOXP3^lo/-^ cells. This is consistent with the expression differences that demonstrated the potential to distinguish between the two populations (**Figure 5B**). When we gated on conventional CD4SPCD25^+^CD127^lo^ cells and assessed FOXP3 expression, the population consisted of 24.5% FOXP3^lo^ cells (range 16.02–29.2%) and 75.6% FOXP3^+/hi^ cells (range 70.10–83%) (**Figure 5D**; representative in **Figure 5A**). However, applying an additional gate for GPA33^+^, TIGIT^+^, PD-1^+^, or CD45RA^+^ together with the conventional strategy increased the proportion of FOXP3^+/hi^ cells to above 90% within each of these subsets (**Figure 5D**; representative **Figure 5C**). Among the four markers tested, GPA33^+^ and TIGIT^+^ cells represented the largest fractions within the conventional Thy-Treg gate (19.87%, range 14.3–26.6% and 20.9%, range 10.9–25.1%, respectively), indicating their suitability for isolating pure mThy-Tregs without compromising yield (**Figure 5E**). In contrast, gating CD27^-^ or CD62L^-^ within the conventional gate enriched the FOXP3^lo^ population. For CD27^-^ cells, FOXP3^lo^ purity exceeded 90%, whereas CD62L^-^ cells reached 76.5% (range 69.7–77.6%) (**Figure 5D**; representative **Figure 5C**). Based on this analysis, CD27^-^ cells—which represent only 2.8% of conventional thymic Tregs (range 2.12–3.75%) (**Figure 5E**)—could be used to isolate a highly pure precursor population.

### Recirculating peripheral Tregs show enhanced antigen experience relative to thymic Tregs and Teffs

**Figure 6:**
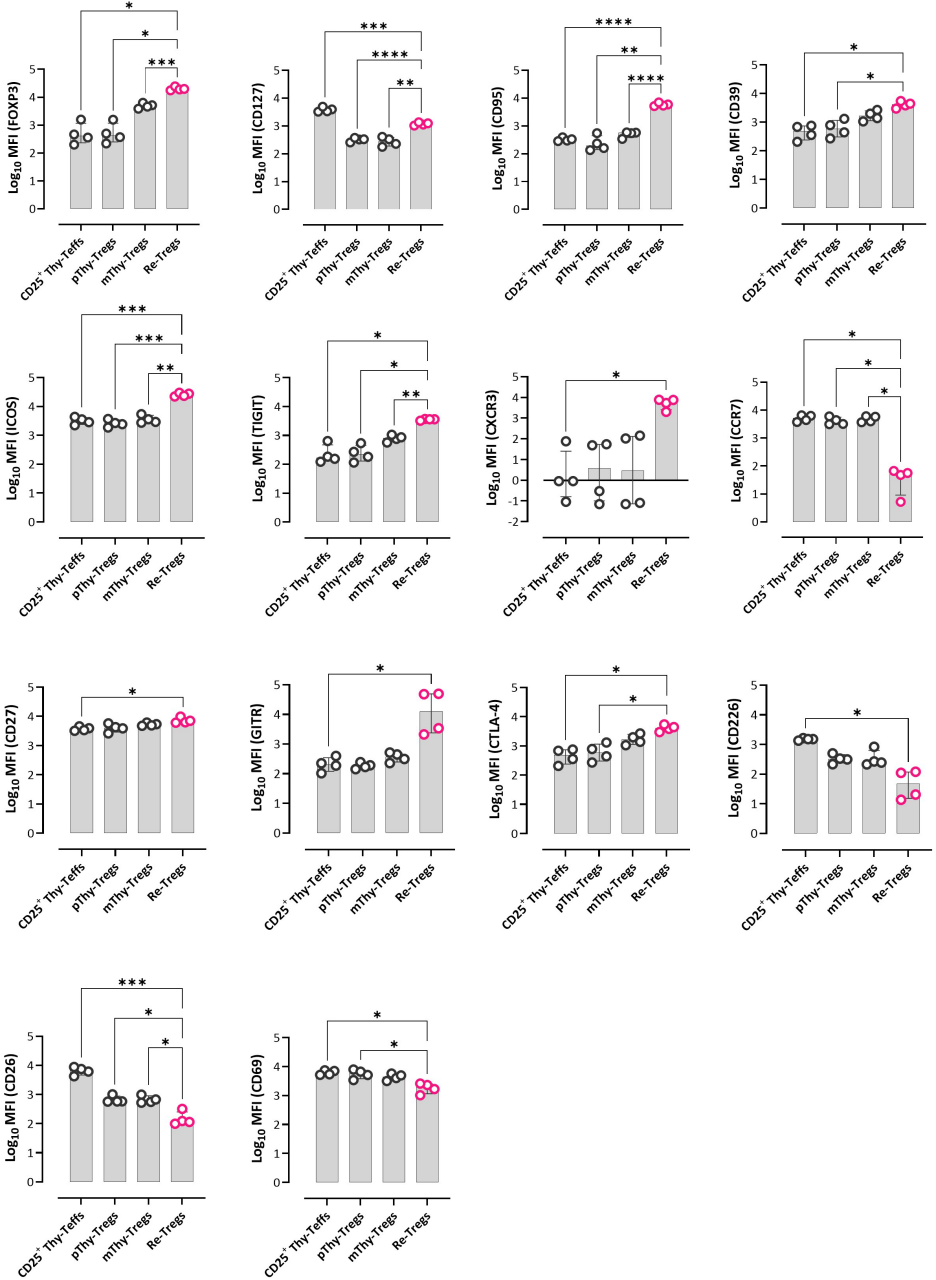
Phenotypic differences between thymic Tregs, Teffs, and recirculating peripheral Tregs. Bar graphs showing the log_10_- transformed MFI values of selected markers within these 4 populations. Data are presented as median with interquartile range (n=4). Statistical analysis was performed using the Kruskal–Wallis test with Dunn’s *post hoc* test and Holm adjustment for multiple comparisons (two-sided, α = 0.05) (*P<0.05, **P<0.01, ***P<0.001, ****P<0.0001). Thy, thymus; pThy-Tregs, precursor thymic Tregs; mThy-Tregs, mature thymic Tregs; Re-Tregs, recirculating peripheral Tregs.

As described earlier, detailed analysis of CD127 and FOXP3 expression within the CD4SP CD25^+^ thymocyte population revealed a small subset defined by the highest FOXP3 expression and intermediate CD127 levels. We hypothesized that this subset represents recirculating peripheral Tregs (Re-Tregs) (**Figure 1C**). As shown in the mean fluorescence intensity (MFI) heatmap (**Supplementary Figure 6A**) and representative plots (**Supplementary Figure 10**), Re-Tregs displayed a distinct phenotype compared to CD25^+^ Thy-Teffs, pThy-Tregs, and mThy-Tregs. Consistent with previous studies (28, 30, 48, 49), Re-Tregs displayed the highest expression of CD95, CD39, ICOS, TIGIT, and CXCR3, along with the lowest expression of CCR7 among all four populations (**Figure 6**). In addition, compared with other thymic Tregs and Teffs, Re-Tregs expressed the highest levels of co-stimulatory receptors (CD27, GITR) and co-inhibitory receptors (CTLA-4), indicating an enhanced activation state (**Figure 6**). Supporting this, Re-Tregs exhibited a memory-like phenotype characterized by elevated CD45RO expression and reduced levels of naïve markers such as CD45RA and CD31 (**Supplementary Figure 6A**). Although Re-Tregs could be mistaken for activated Teffs, the expression of CD226 and CD26—identified in this study as Teff-specific markers—was lowest in this subset, strongly arguing for a subset of the Treg lineage (**Figure 6**). Finally, Re-Tregs showed the lowest expression of the early activation marker CD69 among all four populations, suggesting antigen experience in the periphery before re-migration to the thymus.

## Discussion

Tregs play a crucial role in maintaining immune homeostasis and self-tolerance, making them a promising tool in adoptive cell therapy for treating autoimmune diseases (50), preventing solid organ transplant rejection (51), and managing severe inflammatory (52) and allergic conditions. However, despite the promising potential of Treg-based therapies, early clinical trials have shown variable *in vivo* persistence and efficacy, likely due to differences in source, expansion protocols, and the phenotype of the infused product (2, 53). A major limitation in the human Treg field is the precise identification of these cells, as conventional markers (CD25^+^CD127^lo^FOXP3^+^) are also expressed by activated CD4^+^ Teffs (7). Therefore, examining the phenotypic divergence between Tregs and Teffs beyond conventional markers would be helpful for improving their discrimination. Moreover, this challenge is further compounded by the inherent heterogeneity of Tregs. Most peripheral Tregs have been shown to lose FOXP3 expression and suppressive function under prolonged expansion or pro-inflammatory conditions (54, 55), as demonstrated in both preclinical and clinical studies (56, 57). The extent to which this plasticity arises from Treg heterogeneity or from the conversion of specific Treg subsets into Teffs remains unclear (58).

Over the past two decades, numerous novel markers associated with Treg origin, maturity, stability, and function have been identified (9). Most of these studies, however, have primarily focused on PBMCs as Treg source (10, 11). Due to the increasing therapeutic interest in cord blood-derived Tregs (12–14) and discarded pediatric thymus-derived Tregs (15–17, 20), there is a growing need for a comprehensive direct comparison of Treg populations within these sources. Hence, in this study, we explored naturally occurring therapeutic sources—PBMCs, CBMCs, and thymocytes—to precisely characterize human Tregs and gain deeper insights into their heterogeneity across sources and developmental stages. Our findings may improve Treg isolation protocols by minimizing Teff contamination, provide markers to identify lineage-committed Tregs, and inform which subsets within each source are best suited for genetic engineering in targeted Treg therapies.

In this study, we examined the expression profiles of 31 published markers (9) in Tregs and Teffs (CD25^+^ and CD25^neg^) derived from PBMCs, CBMCs, and thymocytes. Most markers were expressed to some degree in both populations, with none being entirely specific to either. Despite the overlap, the surface markers GPA33 (38, 39) and TIGIT (36, 37), the intracellular CTLA-4 (40, 59), and the transcription factor Helios (41) showed higher expression levels in Tregs than in Teffs across all three sources. Prior studies in PBMCs have shown that co-stimulatory and co-inhibitory receptors, including CTLA-4 and TIGIT, distinguish Tregs from Teffs (10). In line with this study, our data also suggest that CTLA-4 and TIGIT represent robust markers for future Treg sorting or quality control across PBMCs, CBMCs, and thymocytes, given that TIGIT was expressed by less than 5% of Teffs and CTLA-4 by less than 15%. In contrast, a relatively higher proportion of CD25^neg^ Teffs in CBMCs and thymocytes expressed Helios, and a similar proportion of CD25^neg^ Teffs in CBMCs expressed GPA33—unlike PBMCs-derived Teffs, which rarely expressed these markers. These findings suggest that antigen unexperienced T cells may initially express Helios and GPA33 but lose them following antigen exposure in the periphery. Thy-Teffs showed low GPA33 expression, which may reflect either delayed induction or loss after prolonged exposure to self-antigens and activation. Moreover, in our study, Helios, CTLA-4, and TIGIT appeared strongly linked to the Treg developmental lineage, as significantly higher proportions of DP-Tregs, pThy-Tregs, and mThy-Tregs expressed these markers compared with CD25^neg^ Thy-Teffs. This observation supports the previously held notion that Helios marks lineage-committed nTregs (41). However, this interpretation remains debated, given reports of inducible Helios expression in pTregs and the coexistence of Helios-positive and -negative FOXP3^+^ nTregs in humans (60–64). Further functional experiments, particularly using purified Tregs and Teffs, will be required to confirm these findings and strengthen our conclusions.

A higher proportion of Teffs than Tregs expressed the co-stimulatory receptors CD26 and CD226, regardless of source or CD25 expression status (CD25^+^ or CD25^neg^). Over the past decades, considerable effort has been devoted to identifying negative selection markers that enable efficient isolation of pure, highly functional Tregs while minimizing contamination by CD4^+^ Teffs. Our findings highlight CD226 as a strong candidate for such strategies in both research and clinical applications, as its expression was minimal—detected in less than 10% of AB-Tregs and less than 5% of CB-Tregs and Thy-Tregs. Moreover, CD226 was expressed by a higher frequencies of CD25^neg^ Thy-Teffs than of DP-Tregs, pThy-Tregs, and mThy-Tregs. These findings support previous studies showing that exclusion of CD226-expressing cells during Treg sorting enhances the purity, lineage stability, and suppressive functionality of the resulting Treg population (42). In contrast, although CD26 has been proposed in the literature as a negative selection marker for Tregs (43), our data show that >50% of AB- and CB-Tregs and ~20% of Thy-Tregs expressed CD26, limiting its suitability for this purpose.

We compared FOXP3^+^ Tregs across PBMCs, CBMCs, and thymocytes. In PBMCs, CD45RO^+^ cells constituted approximately 57–76% of the Treg population, indicating limited donor-to-donor variability. Moreover, our data also suggest that not all AB-Tregs expressed the key markers associated with Treg function and lineage stability, including CTLA-4, ICOS, Helios, GPA33, and CD31 (9). This finding highlights the heterogeneity of AB-Tregs, comprising distinct subsets with varying functional capacities, underscoring the need to selectively enrich for stable, functional populations to enhance therapeutic efficacy. Miyara et al. have previously subclassified human Tregs based on CD45RA expression (65), and several groups have proposed the use of CD45RA^+^ naïve Tregs from adult peripheral blood for therapeutic applications (66–68) or as a starting population for further engineering (69). However, one major limitation of our study is that the 31 markers analyzed were distributed across six panels. Notably, CD45RA and CD45RO were included in only one panel (Panel 2; **Supplementary Table 2**), restricting subset-specific comparisons to only few markers within this panel. By addressing this limitation in future studies using larger panels enabled by spectral flow cytometry, we can better assess the phenotypic homogeneity of naïve CD45RA^+^ adult Tregs.

In line with previous studies (34, 35), the majority of CBMCs-derived Tregs displayed a naïve phenotype, characterized by CD45RA expression. Furthermore, among all three sources, Tregs derived from CBMCs appeared more homogeneous and likely more stable, supporting their potential suitability for therapeutic applications requiring consistent Treg functionality (13). Over 95% of CB-Tregs expressed Helios, arguing for enhanced lineage stability. The small fraction (~5%) of Helios-negative Tregs in cord blood likely represents peripherally induced Tregs generated during fetal development (70, 71).

Our analysis further revealed marked heterogeneity within Thymocytes, comprising multiple developing Treg and Teff subsets. Although Thy-Treg development has been extensively studied in murine models, the phenotypic heterogeneity and developmental pathways of human Thy-Tregs remain poorly defined. We identified FOXP3^+^ DP-Tregs, consistent with previous studies showing that FOXP3 expression is detectable at the DP stage and that these cells may substantially contribute to the CD4SP thymic Treg pool in humans, in contrast to murine models (22, 72, 73). Furthermore, we identified CD25^+^CD127^lo^FOXP3^lo^CD4SP precursors, which have been reported to acquire FOXP3 expression and mature into functional FOXP3^+^ mThy-Tregs (18). Although thymocytes are generally expected to be predominantly naïve, most mThy-Tregs exhibited a CD45RO^+^ phenotype. Consistent with previous reports (46), we found that CD45RA and GPA33 expression is acquired at later stages of Treg development, as indicated by the lower frequencies and expression levels of these markers in DP-Tregs and pThy-Tregs compared with mThy-Tregs. Notably, between 20–40% of mThy-Tregs expressed CD45RA or GPA33, suggesting that a substantial fraction of mThy-Tregs are not fully mature at the time of harvest.

Analysis of CTLA-4, TIGIT and Helios expression among DP-Tregs, pThy-Tregs, and mThy-Tregs revealed comparable frequencies in DP-Tregs and mThy-Tregs, whereas pThy-Tregs expressed these markers at significantly lower levels. This raises questions about the extent to which pThy-Tregs are committed to bona fide FOXP3^+^ Thy-Tregs and questions the currently used thymic Treg isolation strategy that solely relies on CD25 (15). Consistent with our results, a recent study reported that not all CD4SPCD25^+^FOXP3^lo^ cells differentiate into typical thymus-derived Tregs; some acquire alternative functions or differentiate into other T cell subsets (74). Therefore, for therapeutic purposes, it may be important to exclude these precursor cells. However, because pThy-Tregs differ from mThy-Tregs primarily by FOXP3 expression—and FOXP3 is an intranuclear marker unsuitable for live-cell sorting—conventional isolation methods cannot effectively remove these precursors.

We identified surrogate surface markers capable of distinguishing pThy-Tregs from mThy-Tregs in humans. Specifically, CD45RA/CD45RO, GPA33, TIGIT, and PD-1, when used alongside conventional markers (CD3^hi^CD4^+^CD8^-^CD25^+^CD127^lo^), enabled exclusive discrimination of FOXP3^+/hi^ mThy-Tregs from pThy-Tregs. Because large numbers of Tregs can be isolated from thymocytes, they represent a promising source for future allogeneic off-the-shelf Treg therapies (15). In thymic Tregs, GPA33^+^ and TIGIT^+^ cells each make up about 20% of the total Treg population. Selecting these subsets would still lead to sufficient Treg yield, making them practical candidates for therapeutic use. Moreover, our study also suggests that gating on CD27^-^ cells together with conventional Treg markers enables the isolation of FOXP3^lo^ pThy-Tregs at >90% purity. CD27 has been previously reported to distinguish functional Tregs (75); therefore, it remains unclear whether CD27^-^ pThy-Tregs can differentiate into functional Tregs following *in vitro* expansion. The *in vitro* expansion capacity and phenotypic stability of pThy-Tregs remain poorly characterized. Targeted isolation of the CD27^-^ population, which represents only ~2.8% of total thymic Tregs but yields a highly pure pThy-Treg population, may help clarify the potential benefits and limitations of these precursor cells for therapeutic applications. To our knowledge, this is the first study to report surface marker alternatives for isolating pure mature Thy-Tregs.

Our study also provided a comprehensive phenotypic characterization of recirculating peripheral Tregs within thymocytes. Recent studies have shown that circulating CD25^+^FOXP3^+^ Tregs can migrate back into the thymus (28, 29, 76, 77). We identified a small CD25^+^ population characterized by the highest FOXP3 expression, intermediate CD127 levels, a memory phenotype with brightest CD45RO and CD95 expression, and lack of CD31 expression. This population exhibited strong expression of co-stimulatory and co-inhibitory receptors—including ICOS, TIGIT and CTLA-4—chemokine receptors such as CXCR3, and functional Treg markers such as CD39, consistent with the phenotype of recirculating Tregs described in murine and human studies (29, 46, 49, 76, 78, 79). While this phenomenon is well established in mice, technical limitations have hindered its clarification in humans, where recirculating Tregs are often difficult to distinguish from activated Teffs or locally activated developing Thy-Tregs. In our study, the population identified as recirculating Tregs showed the lowest expression of CD226 and CD26—markers of Teffs—ruling out contamination by activated Teffs. Moreover, these cells exhibited the lowest levels of CCR7 expression, consistent with previous observations (48). Tregs develop in the thymic medulla and CCR7 is essential for the migration of precursors cells from cortex to medulla (46, 48, 80, 81). Therefore, in our study, we excluded the possibility that the highly antigen-experienced, putative recirculating Tregs represented developing Tregs in the thymic medulla. Additionally, the recirculating Tregs have been reported to express CCR6 (48) and to show upregulated expression of CD54, CCR5, and CD2 (30); however, because these markers were not included in our analysis, we were unable to directly compare CCR6 and CCR7 expression or assess these markers within the same dataset. Despite the limitation of the small number of donors tested, our study identified GPA33 as an additional marker that, in combination with conventional markers, can facilitate the isolation of fully developed mThy-Tregs, as neither pThy-Tregs nor recirculating peripheral Tregs expressed this marker.

## Conclusions

Despite the substantial phenotypic overlap between Tregs and Teffs within the CD4^+^ compartment, we identified a concise set of markers expressed at higher frequencies in Tregs than in Teffs (and vice versa), consistent across all three therapeutic Treg sources. Incorporating these markers into standard sorting and quality-control panels can increase isolation purity for research and clinical applications. In thymocytes, we phenotyped Tregs across multiple developmental stages and provided identification markers for a clearly distinguishable subset of recirculating peripheral Tregs. We furthermore identified a set of five surface markers that together with the classic Treg backbone markers enable clean separation of mature thymic Tregs from the CD4SPCD25^+^FOXP3^lo/–^ population often labeled as thymic Treg precursors. Importantly, our data cautiones against assuming that these precursors uniformly mature into CD4SPCD25^+^FOXP3^+/hi^ thymic Tregs and questions the currently used thymic Treg isolation strategy that solely relies on CD25. As interest in allogeneic Treg therapy grows, a deep understanding from rigorous comparison of starting materials is essential for selecting sources that yield stable, high-quality products. Our study sets the stage for improved Treg isolation protocols by minimizing Teff contamination, provides markers to identify lineage-committed Tregs, and facilitates decisions on best suited subsets within each source for genetic engineering in targeted Treg therapies.

## Data Availability

The original contributions presented in the study are included in the article/**Supplementary Material**. Further inquiries can be directed to the corresponding author.

## Supplementary material

Supplementary Figure 1 **Comprehensive gating strategy for PBMCs using flow cytometry.** Sequential manual gating used to identify major T cell populations, followed by delineation of Teff and Treg subsets within the CD4^+^ T cell compartment. CD3^-^CD4^-^ non-T cells (shown in grey) served as the internal negative control. Non-T gating control populations are overlayed in corresponding plots. AB, adult blood.

Supplementary Figure 2 **Comprehensive gating strategy for CBMCs using flow cytometry.** Sequential manual gating used to identify major T cell populations, followed by delineation of Teff and Treg subsets within the CD4^+^ T cell compartment. CD3^-^CD4^-^ non-T cells (shown in grey) served as the internal negative control. Non-T gating control populations are overlayed in corresponding plots. CB, cord blood.

Supplementary Figure 3 **Comprehensive gating strategy for Thymocytes using flow cytometry.** Sequential manual gating used to identify T cell populations at different developmental stages, followed by delineation of Teff and Treg subsets within the CD4SP and CD4CD8DP compartments. CD3^-^CD4^-^CD8^-^ non-T cells (shown in grey) served as the internal negative control. Non-T gating control populations are overlayed in corresponding plots. Thy, thymus; SP, single positive; DP, double positive; pThy-Tregs, precursor thymic Tregs; mThy-Tregs, mature thymic Tregs; Re-Tregs, recirculating peripheral Tregs.

Supplementary Figure 4 **Complete dataset from PBMCs.** (A) Heatmap showing the mean fluorescence intensity (MFI) of the 31 markers analyzed within different Teff and Treg populations. (B) Heatmap displaying the percentage of these populations expressing each of the same 31 markers. Values represent the median from independent donors (n=5). AB, adult blood.

Supplementary Figure 5 **Complete dataset from CBMCs.** (A) Heatmap showing the mean fluorescence intensity (MFI) of the 31 markers analyzed within different Teff and Treg populations. (B) Heatmap displaying the percentage of these populations expressing each of the same 31 markers. Values represent the median from independent donors (n=5). CB, cord blood.

Supplementary Figure 6 **Complete dataset from Thymocytes.** (A) Heatmap showing the mean fluorescence intensity (MFI) of the 31 markers analyzed within different Teff and Treg populations. (B) Heatmap showing the percentage of these populations (only the populations included in the results) expressing each of the 31 markers. Values represent the median from independent donors (n=4). Abbreviations: Thy, thymus; DP, double positive; pThy-Tregs, precursor thymic Tregs; mThy-Tregs, mature thymic Tregs; Re-Tregs, recirculating peripheral Tregs.

Supplementary Figure 7 **Phenotypic differences between developing and mature thymic Tregs and thymic Teffs.** Bar graphs showing the percentage of developing and mature thymic Tregs and Teffs expressing selected markers identified from the phenotypic comparison across PBMCs, CBMCs, and thymocytes. Data are presented as median with interquartile range (n=4). Statistical analysis was performed using the Kruskal–Wallis test with Dunn’s *post hoc* test and Holm adjustment for multiple comparisons (two-sided, α = 0.05) (*P<0.05, **P<0.01, ***P<0.001, ****P<0.0001). Thy, thymus; DP, double positive; pThy-Tregs, precursor thymic Tregs; mThy-Tregs, mature thymic Tregs.

Supplementary Figure 8 **Complete dataset of FOXP3^+^ Tregs.** (A) Heatmap showing the mean fluorescence intensity (MFI) of the 31 markers analyzed within AB-Tregs, CB-Tregs and mThy-Tregs. (B) Heatmap displaying the percentage of these populations expressing each of the same 31 markers. Values represent the median from independent donors (PBMCs and CBMCs: n=5; thymocytes: n=4). AB, adult blood; CB, cord blood; Thy, thymus; mThy-Tregs, mature thymic Tregs.

Supplementary Figure 9 **Phenotypic differences between precursors and mature thymic Tregs.** Overlaid contour plots of FOXP3^+^ mThy-Tregs (pink) and FOXP3^-^/^lo^ pThy-Tregs (grey) showing the expression of the 31 markers analyzed. Marker expression is shown on the x-axis. The five plots highlighted with purple boxes indicate populations suitable for isolating pure FOXP3^+^ mThy-Tregs, whereas the two plots shown in grey indicate populations for isolating pure FOXP3^^-^/lo^ pThy-Tregs. pThy-Tregs, precursor thymic Tregs; mThy-Tregs, mature thymic Tregs.

Supplementary Figure 10 **Phenotypic differences between thymic Tregs, Teffs, and recirculating peripheral Tregs.** Representative heatmap plots showing the mean fluorescence intensity (MFI) of all 31 markers across Teff and Treg subsets within CD25^+^CD4SP cells, identified using the manual gating strategy (**Supplementary Figure 3**). SP, single positive.
