# Supplementary figures and images for: Delineating phenotypic heterogeneity in human regulatory T cells across developmental stages and therapeutic sources
Source: Front Immunol. 2026 Jan 22;17:1697723. doi: 10.3389/fimmu.2026.1697723 (PMC12872549; doi:10.3389/fimmu.2026.1697723)

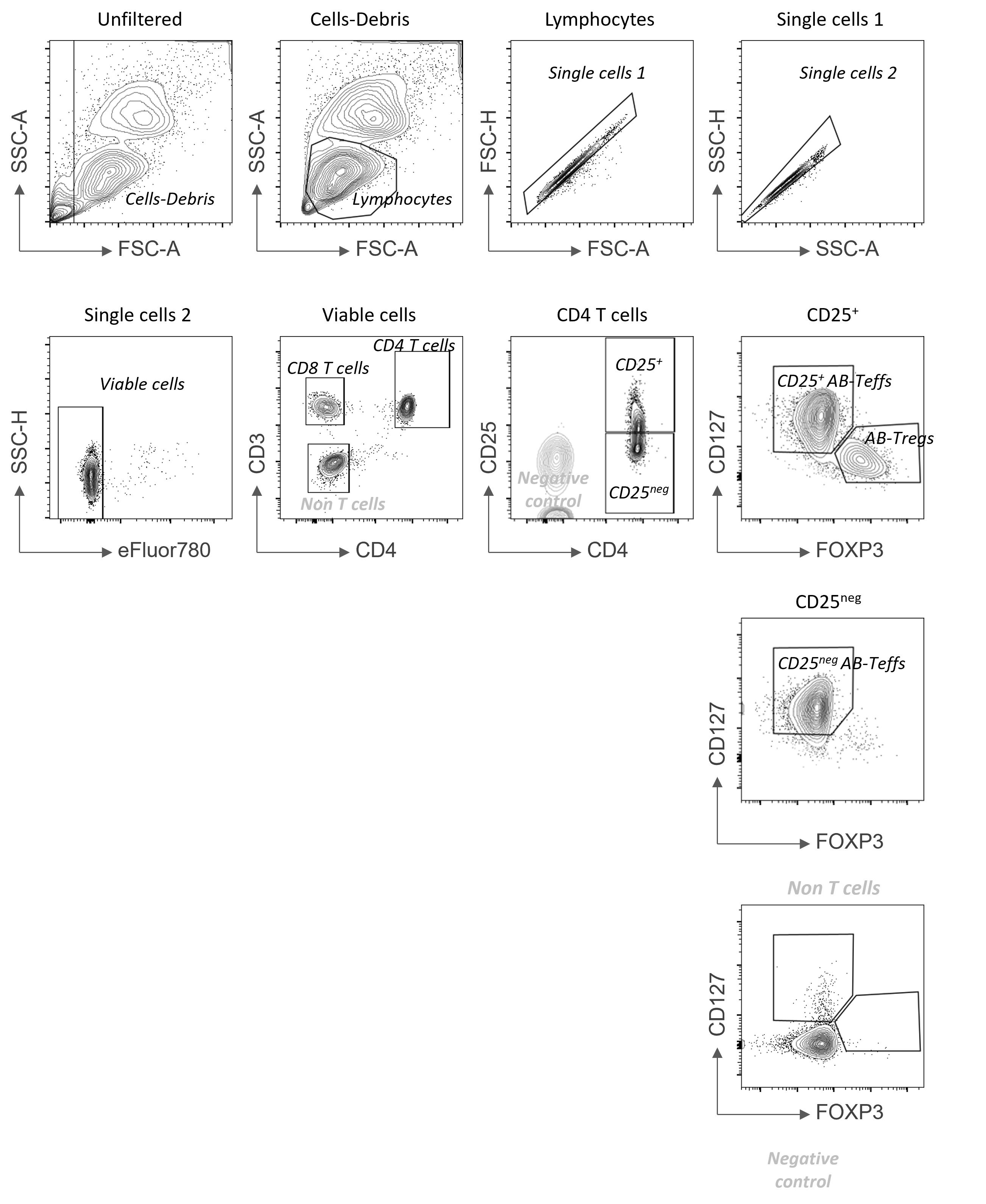

Supplement: Supplementary Figure 1 — Comprehensive gating strategy for PBMCs using flow cytometry. Sequential manual gating used to identify major T cell populations, followed by delineation of Teff and Treg subsets within the CD4+ T cell compartment. CD3-CD4- non-T cells (shown in grey) served as the internal negative control. Non-T gating control populations are overlayed in corresponding plots. AB, adult blood. [file Image1.jpeg]

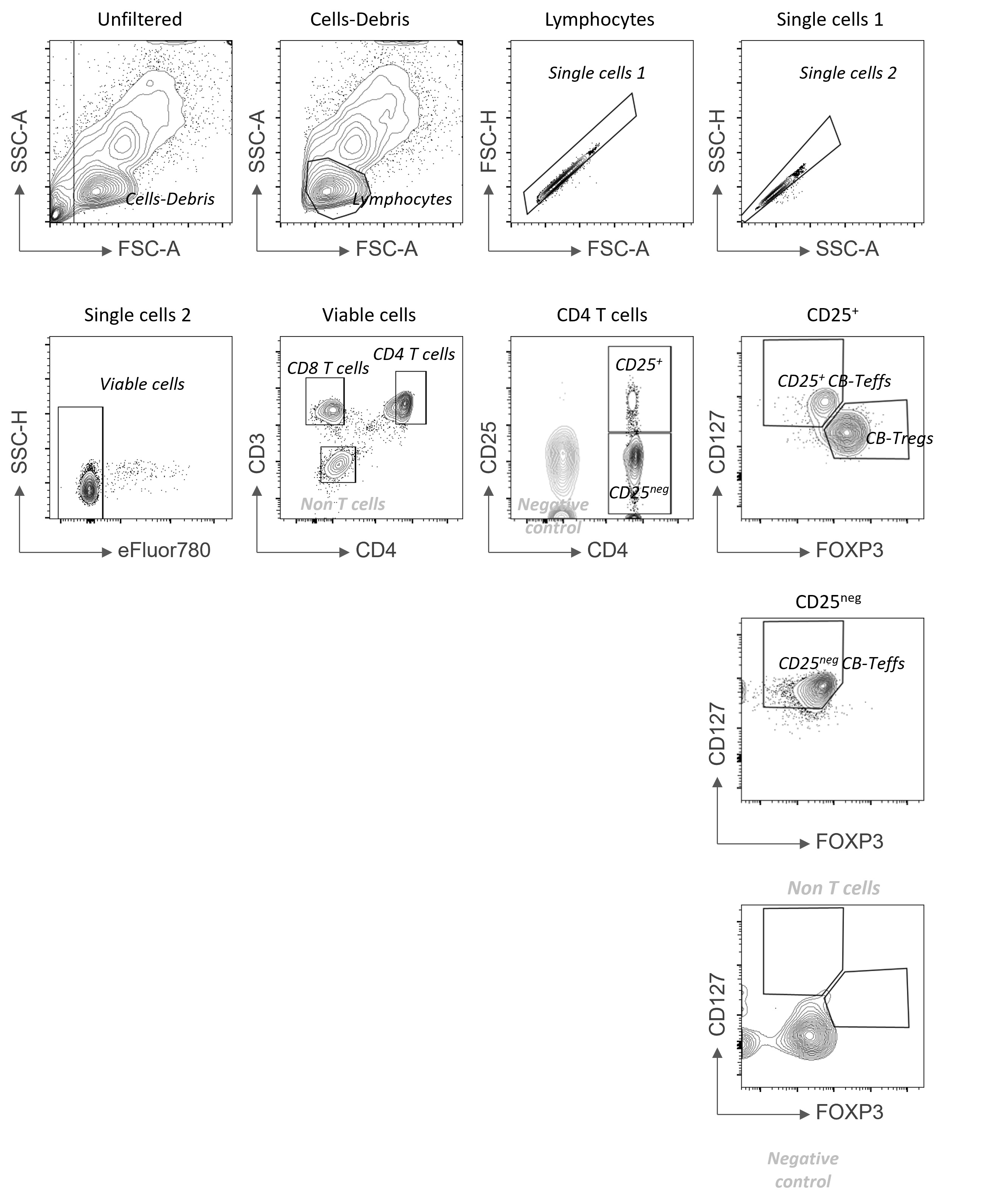

Supplement: Supplementary Figure 2 — Comprehensive gating strategy for CBMCs using flow cytometry. Sequential manual gating used to identify major T cell populations, followed by delineation of Teff and Treg subsets within the CD4+ T cell compartment. CD3-CD4- non-T cells (shown in grey) served as the internal negative control. Non-T gating control populations are overlayed in corresponding plots. CB, cord blood. [file Image2.jpeg]

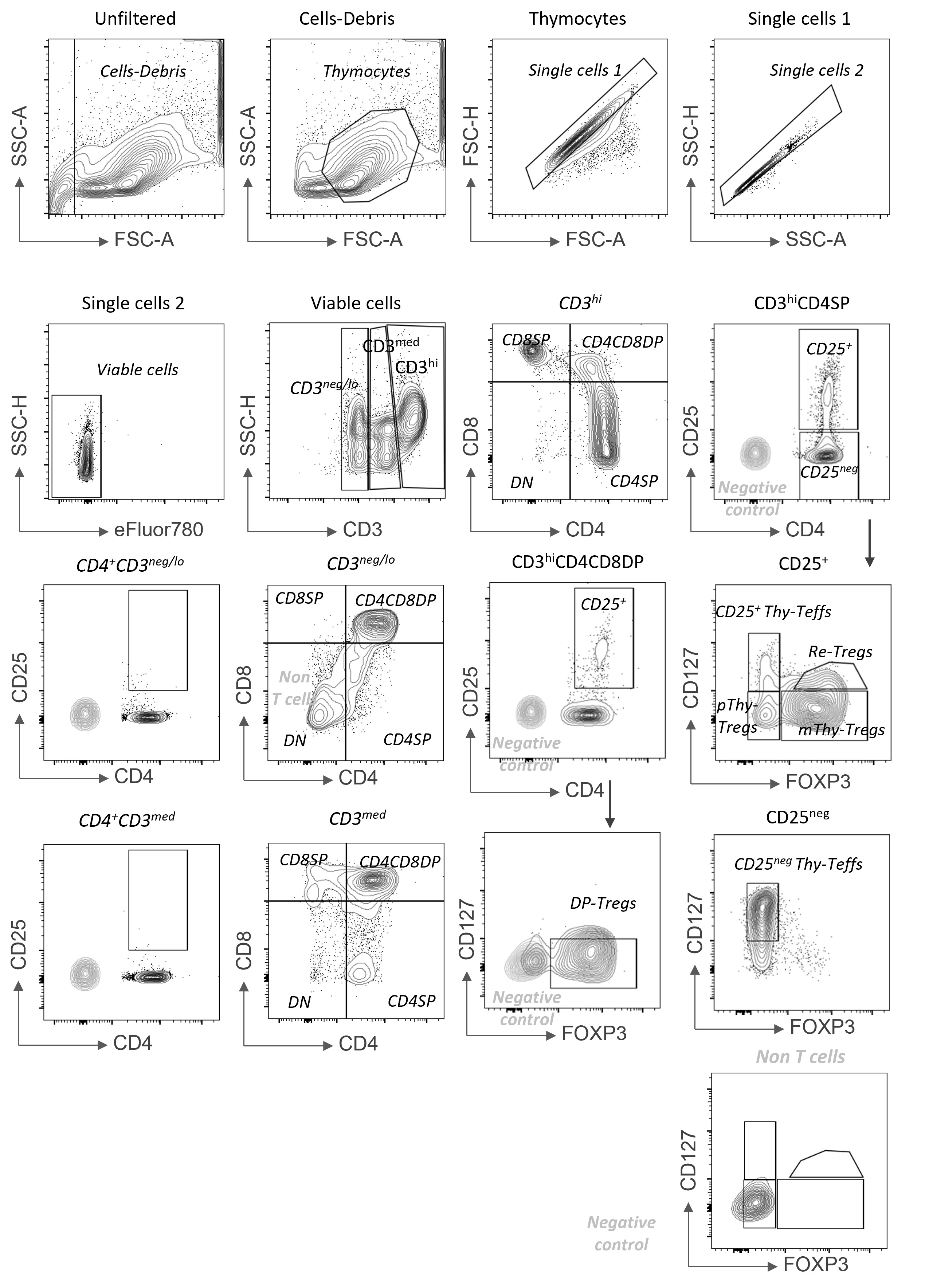

Supplement: Supplementary Figure 3 — Comprehensive gating strategy for Thymocytes using flow cytometry. Sequential manual gating used to identify T cell populations at different developmental stages, followed by delineation of Teff and Treg subsets within the CD4SP and CD4CD8DP compartments. CD3-CD4-CD8- non-T cells (shown in grey) served as the internal negative control. Non-T gating control populations are overlayed in corresponding plots. Thy, thymus; SP, single positive; DP, double positive; pThy-Tregs, precursor thymic Tregs; mThy-Tregs, mature thymic Tregs; Re-Tregs, recirculating peripheral Tregs. [file Image3.jpeg]

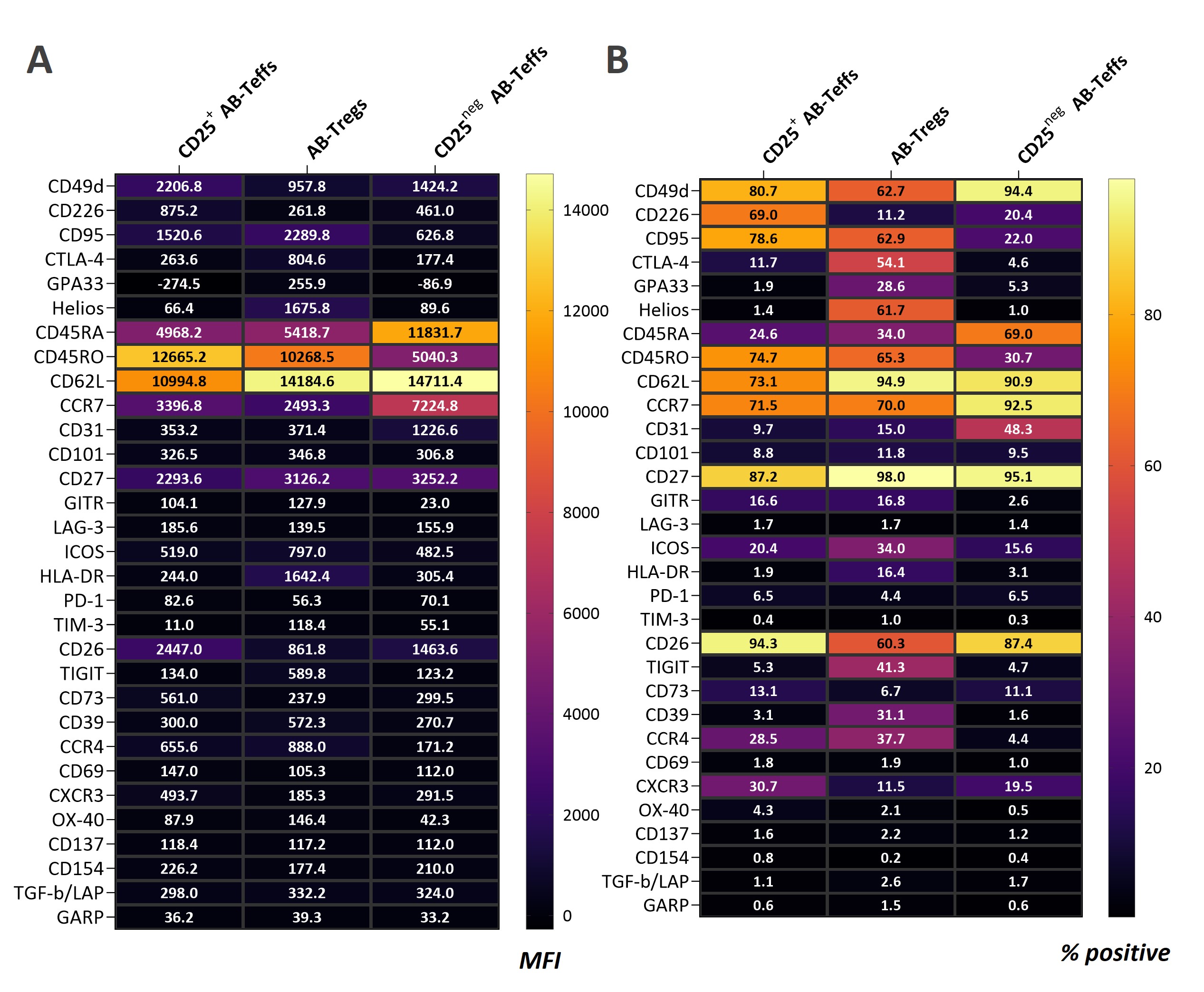

Supplement: Supplementary Figure 4 — Complete dataset from PBMCs. (A) Heatmap showing the mean fluorescence intensity (MFI) of the 31 markers analyzed within different Teff and Treg populations. (B) Heatmap displaying the percentage of these populations expressing each of the same 31 markers. Values represent the median from independent donors (n=5). AB, adult blood. [file Image4.jpeg]

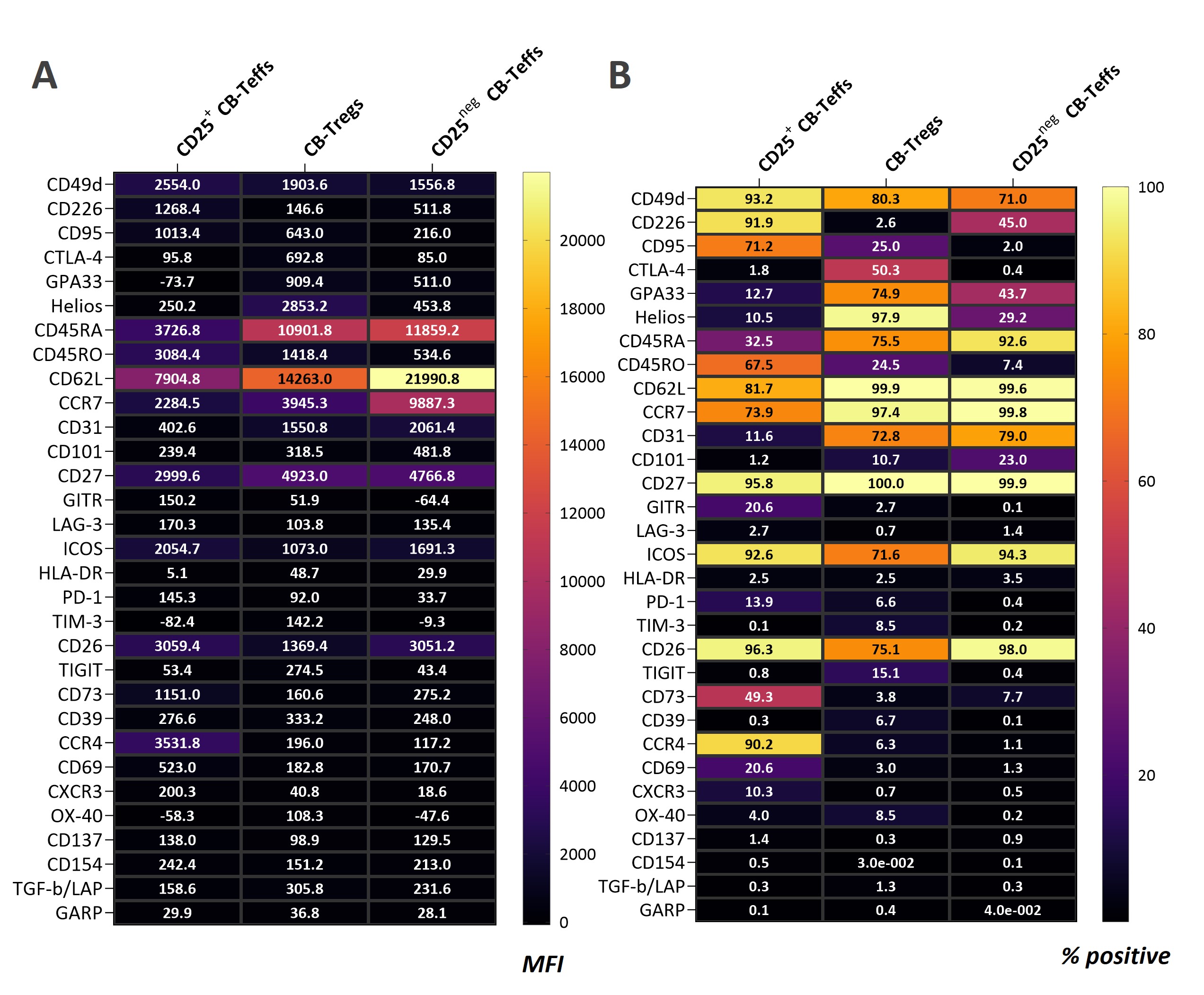

Supplement: Supplementary Figure 5 — Complete dataset from CBMCs. (A) Heatmap showing the mean fluorescence intensity (MFI) of the 31 markers analyzed within different Teff and Treg populations. (B) Heatmap displaying the percentage of these populations expressing each of the same 31 markers. Values represent the median from independent donors (n=5). CB, cord blood. [file Image5.jpeg]

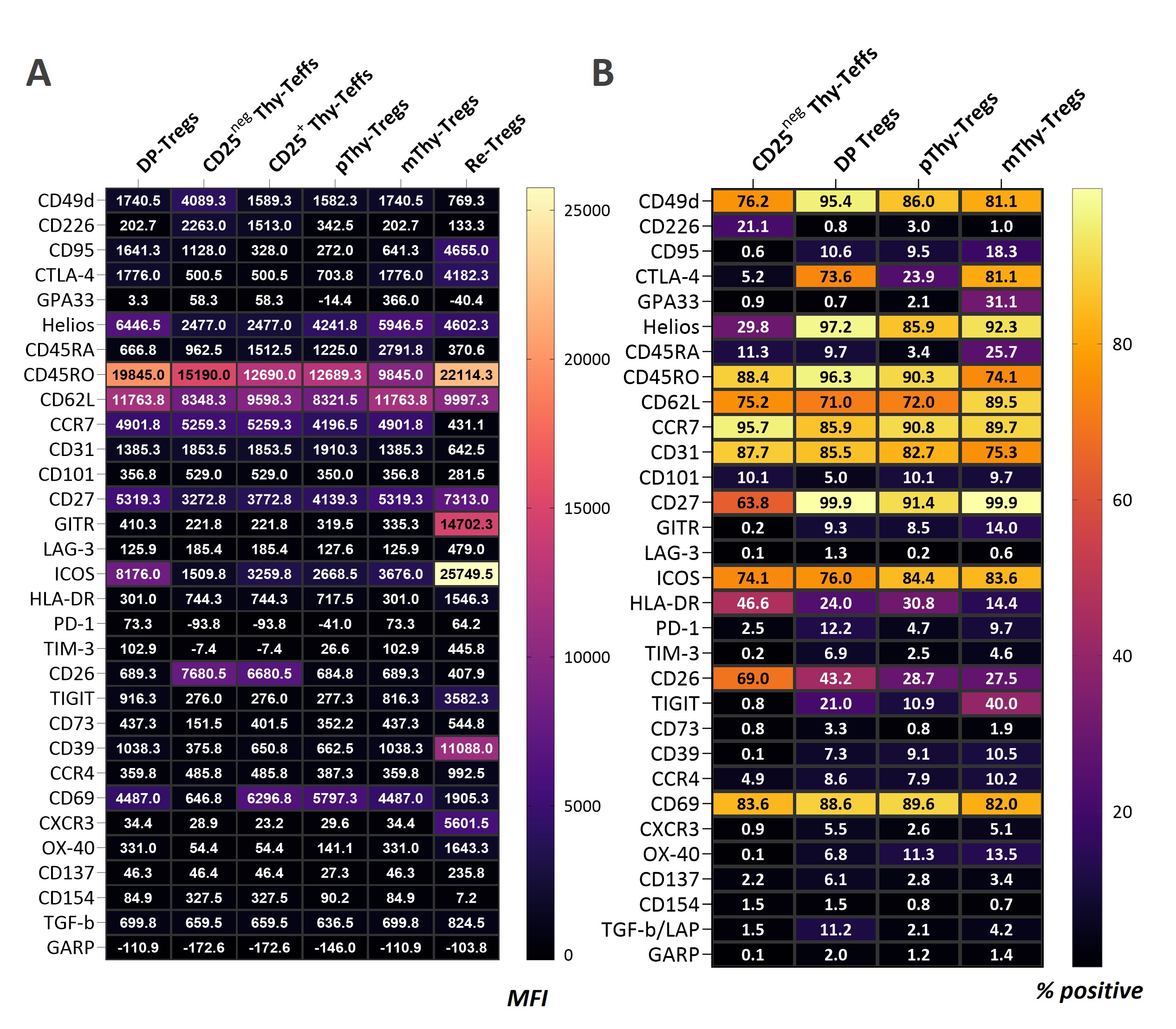

Supplement: Supplementary Figure 6 — Complete dataset from Thymocytes. (A) Heatmap showing the mean fluorescence intensity (MFI) of the 31 markers analyzed within different Teff and Treg populations. (B) Heatmap showing the percentage of these populations (only the populations included in the results) expressing each of the 31 markers. Values represent the median from independent donors (n=4). Abbreviations: Thy, thymus; DP, double positive; pThy-Tregs, precursor thymic Tregs; mThy-Tregs, mature thymic Tregs; Re-Tregs, recirculating peripheral Tregs. [file Image6.jpeg]

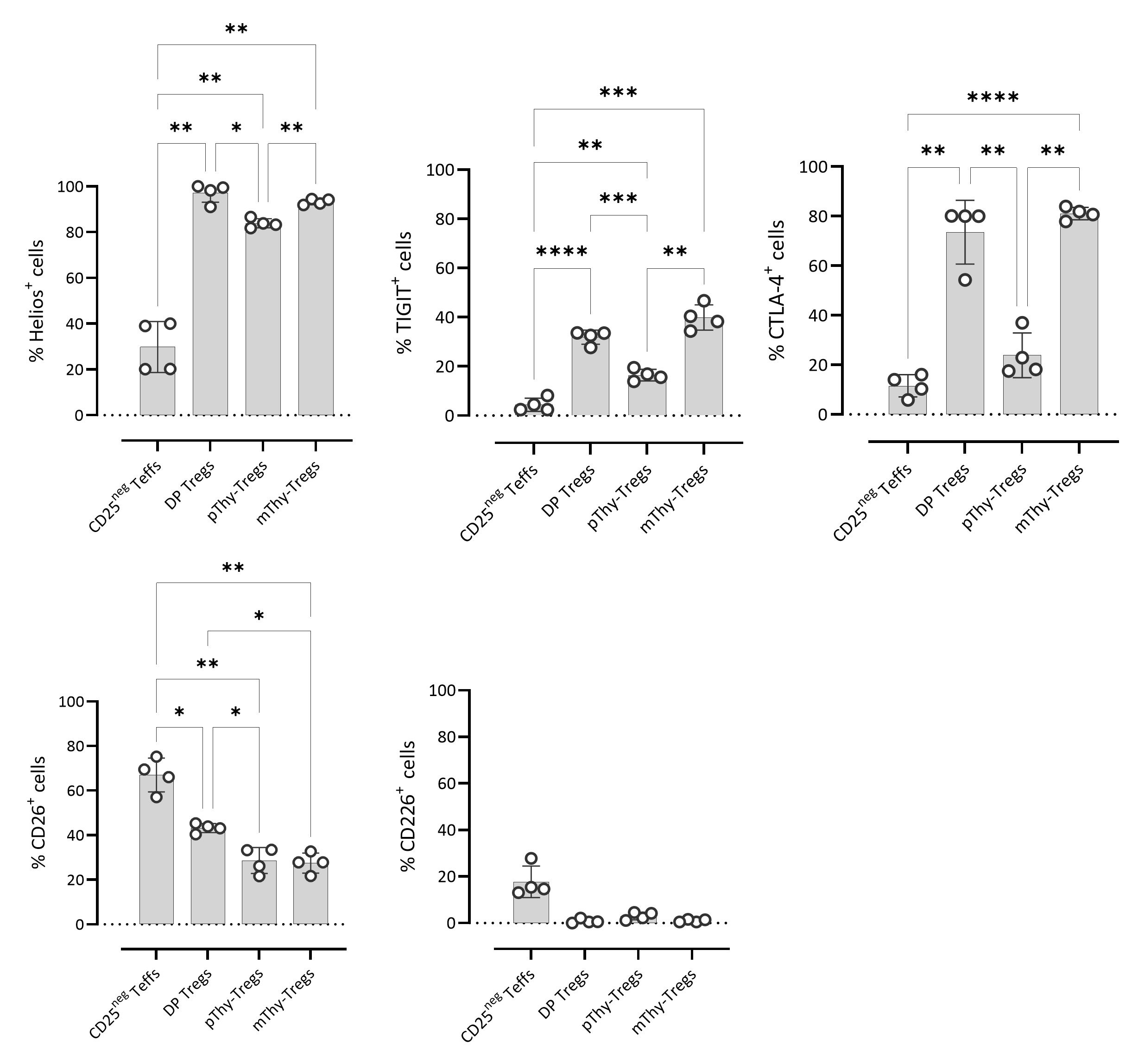

Supplement: Supplementary Figure 7 — Phenotypic differences between developing and mature thymic Tregs and thymic Teffs. Bar graphs showing the percentage of developing and mature thymic Tregs and Teffs expressing selected markers identified from the phenotypic comparison across PBMCs, CBMCs, and thymocytes. Data are presented as median with interquartile range (n=4). Statistical analysis was performed using the Kruskal–Wallis test with Dunn’s post hoc test and Holm adjustment for multiple comparisons (two-sided, α = 0.05) (*P<0.05, **P<0.01, ***P<0.001, ****P<0.0001). Thy, thymus; DP, double positive; pThy-Tregs, precursor thymic Tregs; mThy-Tregs, mature thymic Tregs. [file Image7.jpeg]

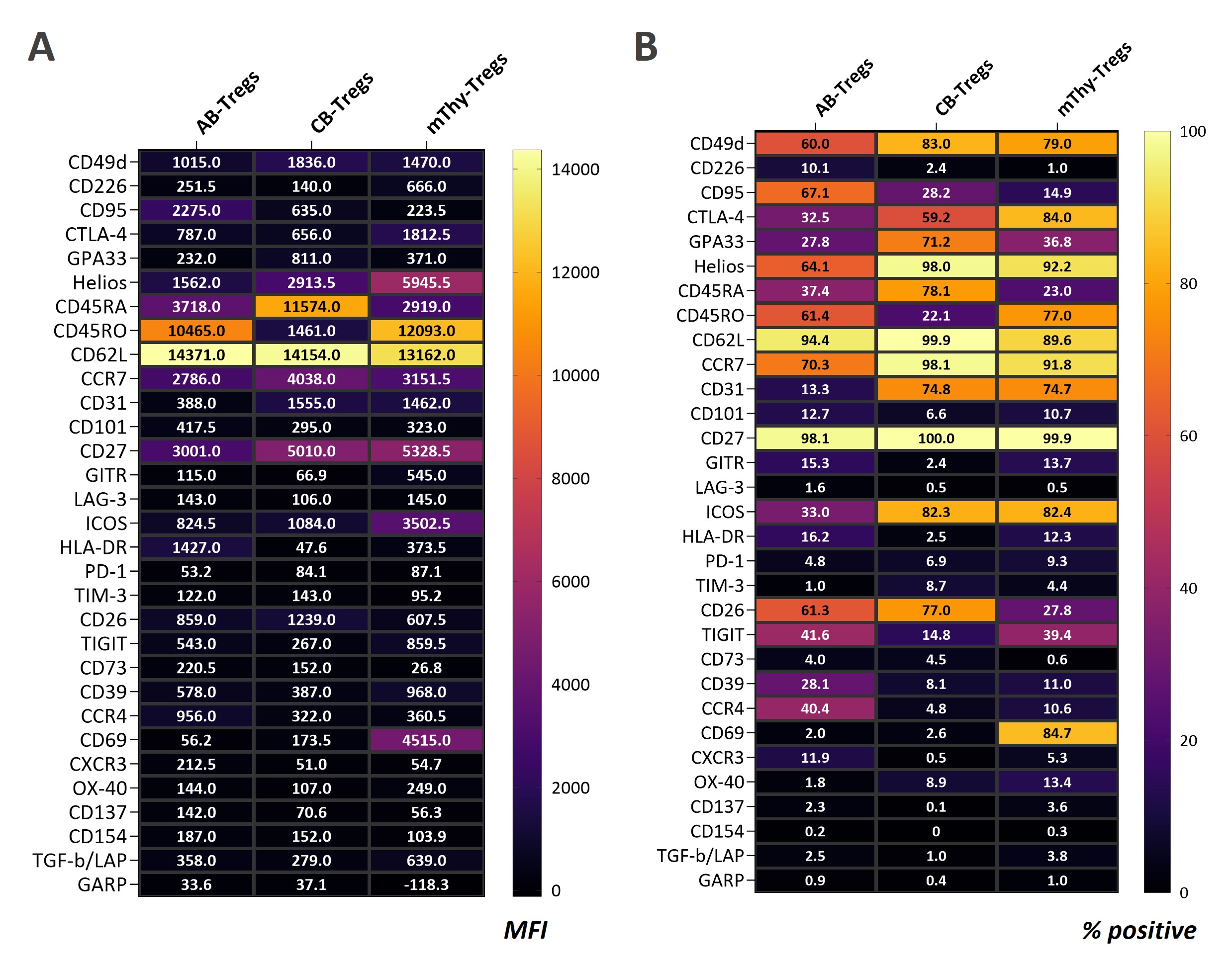

Supplement: Supplementary Figure 8 — Complete dataset of FOXP3+ Tregs. (A) Heatmap showing the mean fluorescence intensity (MFI) of the 31 markers analyzed within AB-Tregs, CB-Tregs and mThy-Tregs. (B) Heatmap displaying the percentage of these populations expressing each of the same 31 markers. Values represent the median from independent donors (PBMCs and CBMCs: n=5; thymocytes: n=4). AB, adult blood; CB, cord blood; Thy, thymus; mThy-Tregs, mature thymic Tregs. [file Image8.jpeg]

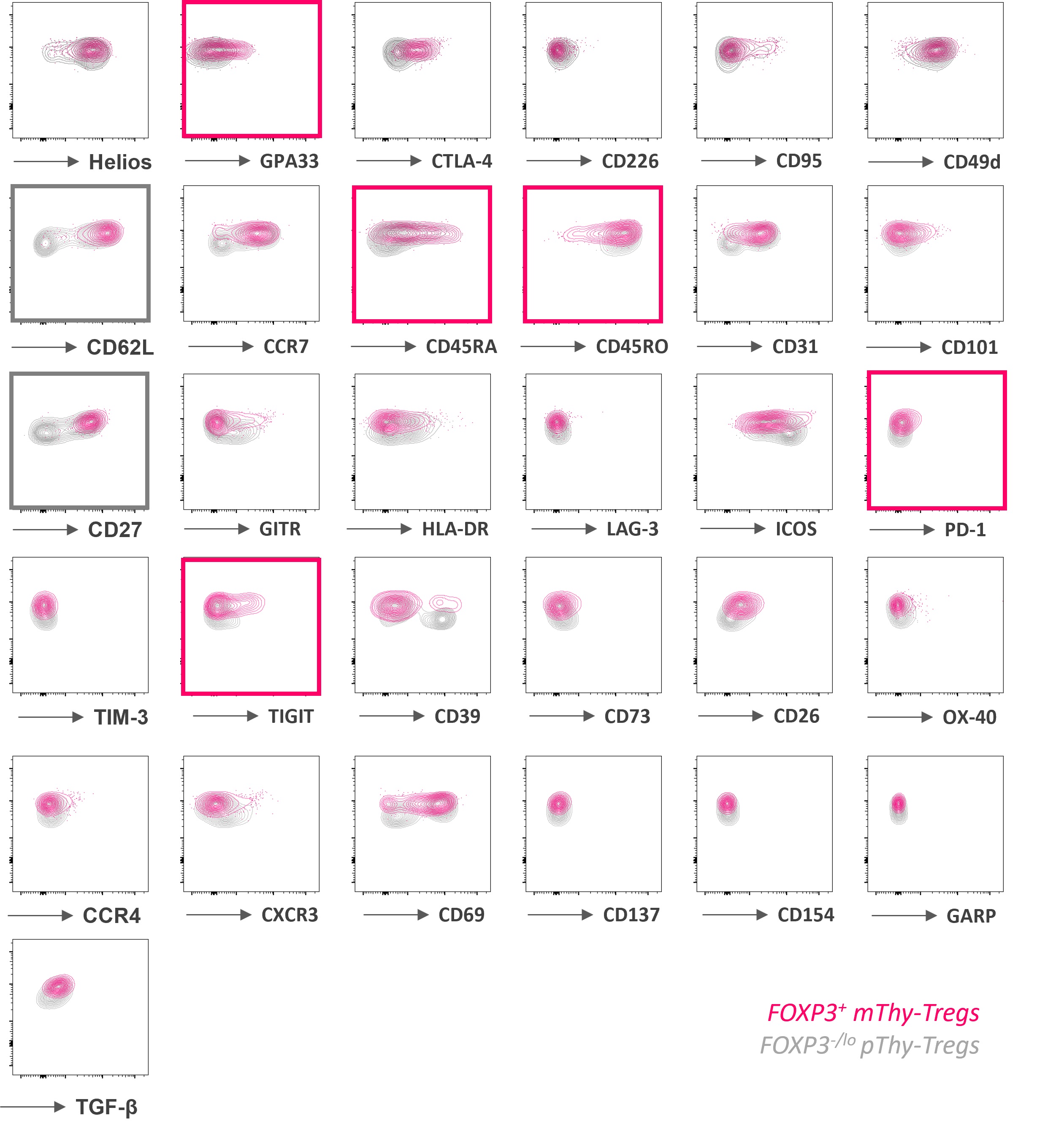

Supplement: Supplementary Figure 9 — Phenotypic differences between precursors and mature thymic Tregs. Overlaid contour plots of FOXP3+ mThy-Tregs (pink) and FOXP3-/lo pThy-Tregs (grey) showing the expression of the 31 markers analyzed. Marker expression is shown on the x-axis. The five plots highlighted with purple boxes indicate populations suitable for isolating pure FOXP3+ mThy-Tregs, whereas the two plots shown in grey indicate populations for isolating pure FOXP3-/lo pThy-Tregs. pThy-Tregs, precursor thymic Tregs; mThy-Tregs, mature thymic Tregs. [file Image9.jpeg]

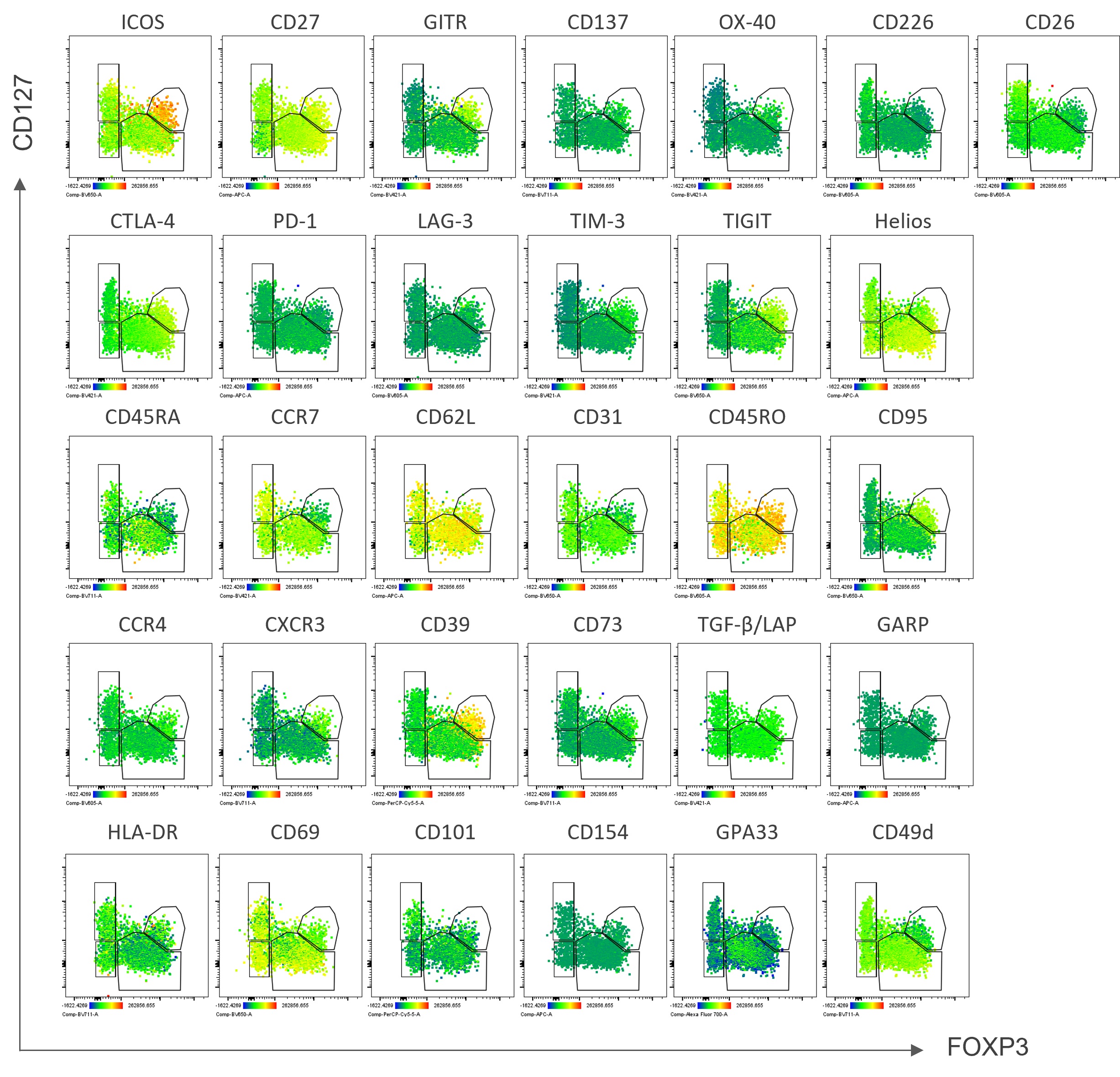

Supplement: Supplementary Figure 10 — Phenotypic differences between thymic Tregs, Teffs, and recirculating peripheral Tregs. Representative heatmap plots showing the mean fluorescence intensity (MFI) of all 31 markers across Teff and Treg subsets within CD25+CD4SP cells, identified using the manual gating strategy (Supplementary Figure 3). SP, single positive. [file Image10.jpeg]
