# Supplementary material for: Delineating phenotypic heterogeneity in human regulatory T cells across developmental stages and therapeutic sources
Source: Front Immunol. 2026 Jan 22;17:1697723. doi: 10.3389/fimmu.2026.1697723 (PMC12872549; doi:10.3389/fimmu.2026.1697723)
Supplement: Supplementary file 11 [file Table1.docx]

**Supplementary Table 1:**

| **Receptor** | **Fluorochrome** | **Clone** | **Titrated volume /million cells** | **Company** |
| --- | --- | --- | --- | --- |
| CD45 | Vioblue | REA747 | 2µl | Miltenyi Biotec |
| 7-AAD | - | **-** | 5µl | Miltenyi Biotec |
| CD3 | FITC | REA A613 | 2µl | Miltenyi Biotec |
| CD4 | BUV395 | RPA-T4 | 2µl | BD Bioscience |
| CD8 | BUV737 | SK1 | 2µl | BD Bioscience |
| CD25 | PE | 4 E3 | 2µl | Miltenyi Biotec |
| CD127 | BV786 | HIL-7R-M21 | 4µl | BD Bioscience |
| FOXP3 | PE-Cy7 | PCH101 | 4µl | Invitrogen, Thermo Fisher Scientific |
| eFluor™ 780 | - | - | 0.2µl | Invitrogen, Thermo Fisher Scientific |
| ICOS/CD278 | BV650 | DX29 | 4µl | BD Bioscience |
| CD27 | APC | M-T271 | 4µl | BD Bioscience |
| GITR/CD357 | BV421 | V27-580 | 2µl | BD Bioscience |
| CD137 | BV711 | 4B4-1 | 4µl | BD Bioscience |
| OX-40/CD134 | BV421 | ACT35 | 4µl | BD Bioscience |
| CD226/DNAM-1 | BV605 | 11A8 | 2µl | BD Bioscience |
| CD26 | BV605 | L272 | 2µl | BD Bioscience |
| CTLA-4/CD152 | BV421 | BNI3 | 2µl | BD Bioscience |
| PD-1/CD279 | APC | MIH4 | 10µl | BD Bioscience |
| LAG-3/CD223 | BV605 | T47-530 | 4µl | BD Bioscience |
| TIM-3/CD366 | BV421 | 7D3 | 1µl | BD Bioscience |
| TIGIT | BV650 | 741182 | 2µl | BD Bioscience |
| CD45RA | BV711 | HI100 | 2µl | BD Bioscience |
| CD45RO | BV605 | UCHL1 | 4µl | BD Bioscience |
| CD62L | APC | 145/15 | 1µl | Miltenyi Biotec |
| CCR7/CD197 | BV421 | 2-L1-A | 2µl | BD Bioscience |
| CD31 | BV650 | WM59 | 4µl | BD Bioscience |
| CD95 | BV650 | DX2 | 2µl | BD Bioscience |
| CCR4/CD194 | BV605 | 1G1 | 4µl | BD Bioscience |
| CXCR3/CD183 | BV711 | 1C6/CXCR3 | 4µl | BD Bioscience |
| CD39 | PerCPCy5.5 | TU66 | 2µl | BD Bioscience |
| CD73 | BV711 | AD2 | 2µl | BD Bioscience |
| TGF-β1 | BV421 | TW4-9E7 | 4µl | BD Bioscience |
| GARP | APC | 7B11 | 4µl | BD Bioscience |
| HLA-DR | BV711 | G46-6 | 2µl | BD Bioscience |
| CD69 | BV650 | FN50 | 4µl | BD Bioscience |
| CD101/BB27 | PerCPCy5.5 | BB27 | 2µl | BioLegend |
| CD154 (CD40L) | BV605 | 24-31 | 4µl | BD Bioscience |
| GPA33 | Alexa Fl700 | 2963D | 4µl | R and D Systems |
| CD49d | BV711 | 9F10 | 4µl | BD Bioscience |
| Helios | APC | 22F6 | 2µl | BioLegend |

*Intracellular/intranuclear markers
