# Supplementary material for: Delineating phenotypic heterogeneity in human regulatory T cells across developmental stages and therapeutic sources
Source: Front Immunol. 2026 Jan 22;17:1697723. doi: 10.3389/fimmu.2026.1697723 (PMC12872549; doi:10.3389/fimmu.2026.1697723)
Supplement: Supplementary file 12 [file Table2.docx]

**Supplementary table 2:**

|  |  | **Backbone panel** | **Panel-1** | **Panel-2** | **Panel-3** | **Panel-4** | **Panel-5** | **Panel-6** |
| --- | --- | --- | --- | --- | --- | --- | --- | --- |
| UV | BUV 737 | **CD8** | **CD8** | **CD8** | **CD8** | **CD8** | **CD8** | **CD8** |
| 355 | DAPI |  |  |  |  |  |  |  |
|  | BUV395 | **CD4** | **CD4** | **CD4** | **CD4** | **CD4** | **CD4** | **CD4** |
| Violet | BV786 | **CD127** | **CD127** | **CD127** | **CD127** | **CD127** | **CD127** | **CD127** |
| 405 | BV711 |  | CD49d | CD45RA | HLA-DR | CD73 | CXCR3 | CD137 |
|  | BV650 |  | CD95 | CD31 | ICOS | TIGIT | CD69 |  |
|  | BV605 |  | CD226 | CD45RO | LAG-3 | CD26 | CCR4 | CD154 |
|  | BV510 |  |  |  |  |  |  |  |
|  | BV 421 |  | CTLA-4 | CCR7 | GITR | TIM-3 | OX-40 | TGF-β1 |
| Blue | PerCPCy 5.5 |  |  | CD101 |  | CD39 |  |  |
| 488 | FITC | **CD3** | **CD3** | **CD3** | **CD3** | **CD3** | **CD3** | **CD3** |
|  | SCC |  |  |  |  |  |  |  |
| Yellow-Green | PE-Cy7 | **FOXP3** | **FOXP3** | **FOXP3** | **FOXP3** | **FOXP3** | **FOXP3** | **FOXP3** |
| 561 | PE-Cy5 |  |  |  |  |  |  |  |
|  | PE-Texas Red |  |  |  |  |  |  |  |
|  | PE | **CD25** | **CD25** | **CD25** | **CD25** | **CD25** | **CD25** | **CD25** |
| Red | APC-Cy7 | **Viability (eFluor™ 780)** | **Viability (eFluor™ 780)** | **Viability (eFluor™ 780)** | **Viability (eFluor™ 780)** | **Viability (eFluor™ 780)** | **Viability (eFluor™ 780)** | **Viability (eFluor™ 780)** |
| 640 | Alexa Fl700 |  | GPA33 |  |  |  |  |  |
|  | APC |  | Helios | CD62L | CD27 | PD-1 |  | GARP |
