# Supplementary material for: Delineating phenotypic heterogeneity in human regulatory T cells across developmental stages and therapeutic sources
Source: Front Immunol. 2026 Jan 22;17:1697723. doi: 10.3389/fimmu.2026.1697723 (PMC12872549; doi:10.3389/fimmu.2026.1697723)
Supplement: Supplementary file 13 [file Table3.docx]

**Supplementary Table 3:**

| 1 | Co-stimulatory receptors | ICOS, CD27, GITR, CD137, OX-40, CD226, CD26 |
| --- | --- | --- |
| 2 | Co-inhibitory receptors | CTLA-4, PD-1, LAG-3, TIM-3, TIGIT |
| 3 | Naïve markers | CD45RA, CD62L, CCR7, CD31 |
| 4 | Memory markers | CD45RO, CD95 |
| 5 | Chemokine receptors | CCR4, CXCR3 |
| 6 | Markers associated with Treg function | CD39, CD73, TGF-β/LAP, GARP |
| 7 | Treg/Tcell activation markers | HLA-DR, CD69, CD101, CD154 |
| 8 | Transcription factor | Helios |
| 9 | Other surface glycoprotein and integrin | GPA33, CD49d |
